# A Greener, Efficient Approach to Michael Addition of Barbituric Acid to Nitroalkene in Aqueous Diethylamine Medium

**DOI:** 10.3390/molecules19011150

**Published:** 2014-01-17

**Authors:** Hany J. Al-Najjar, Assem Barakat, Abdullah M. Al-Majid, Yahia N. Mabkhot, Manuel Weber, Hazem A. Ghabbour, Hoong-Kun Fun

**Affiliations:** 1 Department of Chemistry, College of Science, King Saud University, P. O. Box 2455, Riyadh 11451, Saudi Arabia; E-Mails: hany_33@hotmail.com (H.J.A.-N.); amajid@ksu.edu.sa (A.M.A.-M.); yahia@ksu.edu.sa (Y.N.M.); 2 Department of Chemistry, Faculty of Science, Alexandria University, P.O. Box 426, Ibrahimia- 21321 Alexandria, Egypt; 3 Department of Chemistry, University of California, Berkeley, CA 94720, USA; E-Mail: manuelweber@berkeley.edu; 4 Department of Pharmaceutical Chemistry, College of Pharmacy, King Saud University, P. O. Box 2457, Riyadh 11451, Saudi Arabia; E-Mails: ghabbourh@yahoo.com (H.A.G.); hfun.c@ksu.edu.sa (H.-K.F.)

**Keywords:** Michael reactions, barbituric acid, aqueous media, green chemistry

## Abstract

An efficient method for the synthesis of a variety of pyrimidine derivatives **3a**–**t** by reaction of barbituric acids **1a**,**b** as Michael donor with nitroalkenes **2a**–**k** as Michael acceptor using an aqueous medium and diethylamine is described. This 1,4-addition strategy offers several advantages, such as using an economic and environmentally benign reaction media, high yields, versatility, and shorter reaction times. The synthesized compounds were identified by ^1^H-NMR, ^13^C-NMR, CHN, IR, and MS. The structure of compound **3a** was further confirmed by single crystal X-ray structure determination.

## 1. Introduction

The Michael reaction is one of the most powerful tools for the formation of carbon–carbon bonds in organic synthesis [1,2,3,4,5,6]. The addition of various active methines compounds to nitroalkenes has received increased attention since the conjugated addition products are aliphatic nitro compounds. These are recognized as versatile synthetic building blocks which can be either transformed into biologically active compounds such as tetrahydropyrans, amino acids, pyrrolidines and lactones [7,8,9], or readily converted into other functionalities such as ketones, amines, carboxylic acids, nitrile oxides, *etc.* [10,11,12,13]. Extensive studies have been devoted to the development of catalytic systems for Michael additions of various active methines such as pronucleophiles to nitroalkenes including cinchona organocatalysts [14], enzymes [15], various chiral amines [16,17], transition metal-free organocatalysts [18,19,20] and chiral metal complexes [21,22,23,24,25].

Recently, the utilization of water as a solvent has emerged as an extensively investigated topic in organic transformations for its environmental friendly character, low cost and properties conferring unique selectivity and reactivity [26]. For these reasons, the development of synthetically useful reactions that take place in water is of considerable topical interest.

Barbituric acids constitute an interesting family of pyrimidinetrione heterocycles [27,28,29]. They are well-known in medicinal chemistry as sedatives, hypnotics, anticonvulsants and anxiolytic agents [30,31,32]. Barbituric acids are also particularly utilized as nucleophiles. In continuation of our research program [33,34,35,36,37], we have investigated the reaction of barbituric acids, as nucleophiles with nitroalkenes as Michael acceptors in water using diethylamine to afford multifunctional pyrimidine systems for biological and pharmacological evaluation. To the best of our knowledge, this is the first successful method of this type using aqueous diethylamine as reaction medium.

## 2. Results and Discussion

We initiated our investigations by using 1,3-dimethylbarbituric acid (**1a**) as C-based nucleophile and nitroalkene (**2a**) as a Michael acceptor for the 1,4-addition strategy in the presence of aqueous diethylamine at ambient temperature, as shown in Table 1. These are the optimal reaction conditions for the construction of Michael product by this strategy [38].

As expected, the Michael product **3a** was obtained in quantitative yield after 1 h (Table 1, entry 1). In addition, other secondary amines were examined. It was found that *i*Pr_2_NH generated the Michael product in a significant yield of 85% (Table 1, entry 2). Additionally, (cyclohexyl)_2_NH and morpholine afforded the product in substantial yields of 82% and 78%, respectively (Table 1, entries 3, 4). NaOH was also examined and was found to be less efficient in the reaction. Only a moderate yield was obtained (Table 1, entry 5). In the absence of amine (entry 6) or water (entry 7), the reaction either could not be performed or proceeded very slowly. The best results, with respect to yield, were obtained by performing the reaction with the combined promoting effects of both H_2_O and Et_2_NH.

**Scheme 1:**
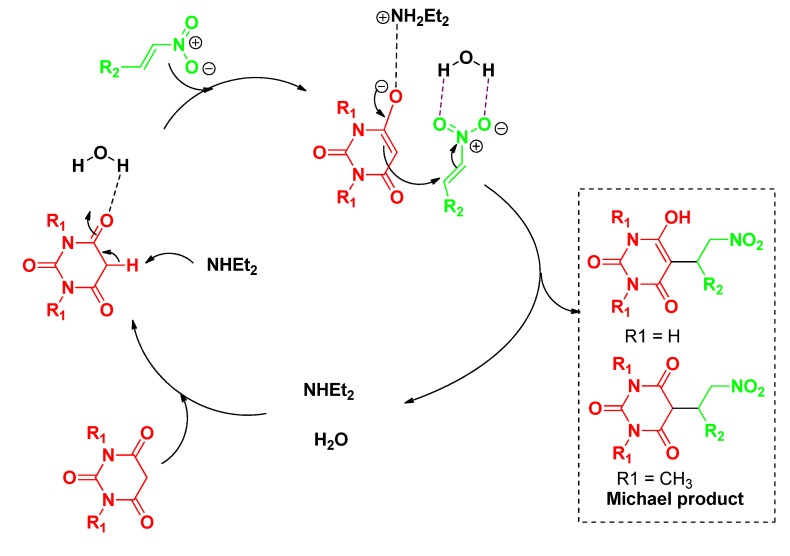
A possible mechanistic pathway.

It is well known that the rate for organic reactions conducted in aqueous media require either hydrogen bonding activation of functional groups by water or the repulsive hydrophobic interactions of the reactants [39,40,41]. We thus envisioned that hydrogen bonding activation in the presence of amine as a base generates an enolate, the catalyst of choice for the Michael strategy (Scheme 1).

**Table 1:** Screening of conditions for the Michael addition reaction of the model substrate *^a^*. 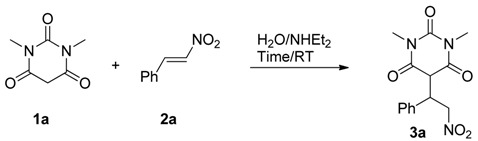

Entry	Condition	Time	Yield (%) *^b^*
1	Et_2_NH/H_2_O	1	99
2	iPr_2_NH/H_2_O	4	85
3	(Cyclohexyl)_2_NH/H_2_O	4	82
4	Morpholine/H_2_O	3	78
5	NaOH/H_2_O	6	65
6	Et_2_NH	10	10
7	H_2_O	10	0

*^a^* All reactions were carried out with 1,3-dimethylbarbituric acid **1a** (1.5 mmol), nitroalkene **2a** (1.5 mmol) and amine (1.5 mmol) in water (1.5 mL) for the specified time. *^b^* Yield of isolated product.

With the optimal reaction conditions in hand, the substrate scope was then investigated. First, 1,3-dimethylbarbituric acid (**1a**) as C-based nucleophile was reacted with eight different phenyl-type substituted nitroalkenes as Michael acceptors (Table 2, entries 1–8). The reactions proved to work well with a range of nitroalkenes bearing either electron-withdrawing or electron-donating groups that produced the desired products with excellent yields (88%–99%).

**Table 2:** Michael addition reaction of barbituric acid derivatives **1** to nitroolefin **2** catalyzed by Et_2_NH in water at room temperature ^*a*^. 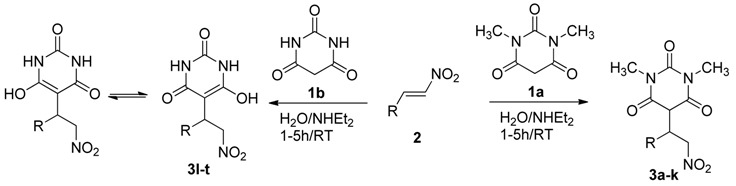

Entry	**3**	R	Yield (%) *^b^*
1	**3a**	Ph	99
2	**3b**	*p*-CH_3_Ph	96
3	**3c**	*p*-BrPh	92
4	**3d**	*p*-ClPh	91
5	**3e**	2,4-Cl_2_Ph	90
6	**3f**	2,6-Cl_2_Ph	91
7	**3g**	*p*-CH_3_OPh	89
8	**3h**	*p*-NO_2_Ph	88
9	**3i**	Ferrocene	93
10	**3j**	CH_3_	96
11	**3k**	Thiophene	95
12	**3l**	Ph	97
13	**3m**	*p*-CH_3_Ph	94
14	**3n**	*p*-BrPh	88
15	**3o**	*p*-ClPh	89
16	**3p**	2,4-Cl_2_Ph	85
17	**3q**	2,6-Cl_2_Ph	86
18	**3r**	*p*-CH_3_OPh	88
19	**3s**	Ferrocene	92
20	**3t**	*p*-NO_2_Ph	87

*^a^* All reactions were carried out with barbituric acid **1** (1.5 mmol), nitroalkene **2** (1.5 mmol) and amine (1.5 mmol) in water (1.5 mL) for the specified time. *^b^* Yield of isolated product.

Next, we applied the conditions to reactions of barbituric acid (**1b**) with eight different phenyl-type substituted nitroalkenes. Interestingly, the Michael product was separated in enol form and not in keto form in very good to excellent yields (Table 2, entries 13–20). Finally, to show the generality of the procedure, we tested ferrocene nitroalkene as Michael acceptor and the products were obtained in very good yields (Table 2, entries 9, 19). A further development has also been achieved by our group. Thus, Michael addition of barbiturate to either aliphatic nitroalkenes or heteroaromatic nitroalkenes affords the pyrimidine adduct (Table 2, entries 10, 11) with excellent yields (96% and 95%, respectively). Thus, the methodology not only suitable for aromatic nitroalkenes, but also aliphatic and heteroaromatic nitroalkenes.

**Figure 1:**
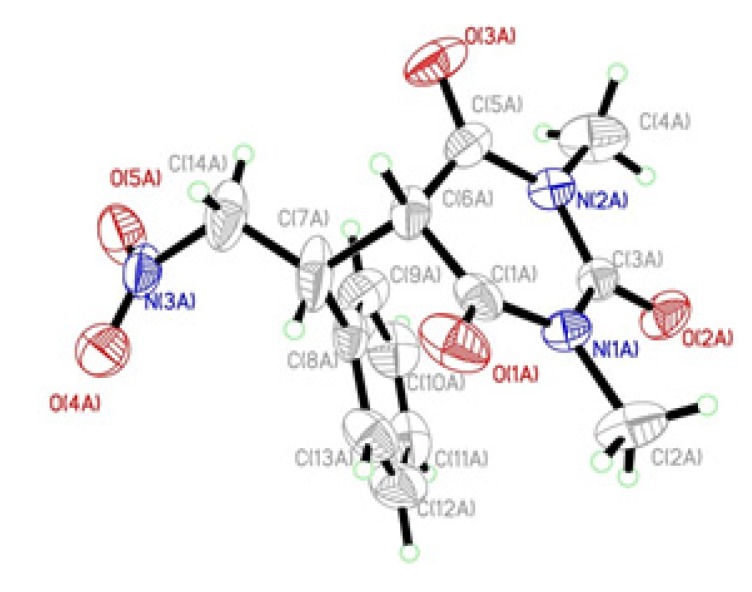
ORTEP representation of the structure of **3a**.

The X-ray structure of **3a** (Figure 1) was obtained by single crystal structure determination from a single crystal grown from CHCl_3_/Et_2_O as a solvent. The structure shows interesting characteristics and also the hydrogen-bonding interactions are listed (see Supporting Information). The packing of the molecules in the crystal structure is stabilized by C–H⋅ ⋅ ⋅O hydrogen bonds into a three-dimensional framework structure.

## 3. Experimental

### 3.1. General Information

All the chemicals were purchased from Sigma-Aldrich and Fluka (Seelze, Germany), and were used without further purification unless otherwise stated. All melting points were measured on a Gallenkamp melting point apparatus in open glass capillaries and are uncorrected. IR Spectra were measured as KBr pellets on a Nicolet 6700 FT-IR spectrophotometer (Madison, WI, USA). The NMR spectra were recorded on a Varian Mercury Jeol-400 NMR spectrometer (Tokyo, Japan).^1^H-NMR (400 MHz), and ^13^C-NMR (100 MHz) were run in either deuterated dimethylsulphoxide (DMSO-*d_6_*) or deuterated chloroform (CDCl_3_). Chemical shifts (*δ*) are referred in terms of ppm and *J*-coupling constants are given in Hz. Mass spectra were recorded on a Jeol JMS-600 H instrument (Tokyo, Japan). Elemental analysis was carried out on a Perkin Elmer 2400 Elemental Analyzer (Vernon Hills, Illinois, IL, USA); CHN mode.

### 3.2. General Procedure for Synthesis of Nitroalkenes ***2a–k***

Equimolar amounts of aryl aldehyde and nitromethane (1 equiv.) were dissolved in 95% ethyl alcohol (100 mL) at room temperature and then a solution of sodium hydroxide (1 equiv.) in ethyl alcohol (30 mL) was added from a dropping funnel at a rate of 5 mL per minute. The solution of the nitromethane and aldehyde in alcohol was then vigorously stirred at room temperature for 1 h. As the reaction proceeded, the insoluble sodium salt of the condensation product precipitated. After all of the alkali had been added and with the temperature kept below 20 °C, ice water (about 30 mL) was slowly added until the precipitate just dissolved. This clear, cold solution was slowly added to a solution of 15% HCl (50 mL) and more cold water (300 mL) was added. A fine, light yellow precipitate was immediately formed and filtered with suction after standing for half an hour. The product thus formed was quite pure and was found to be satisfactory as a starting product for subsequent preparations without further purification.

### 3.3. General Procedure for Michael Addition for the Synthesis of ***3a–t***

A mixture of nitroalkene **2a–k** (1.5 mmol), barbituric acid derivatives **1a,b** (1.5 mmol), and Et_2_NH (1.5 mmol) in degassed H_2_O (1.5 mL) was stirred at room temperature for 1 h until TLC showed complete disappearance of the reactants. The product either precipitated or was in an oily form. The reaction mixture was acidified using 1M HCl extract with DCM/10% EtOH then washed with brine and dried over MgSO_4_ to afford **3a–t**.

*1,3-Dimethyl-5-(2-nitro-1-phenylethyl)pyrimidine-2,4,6(1H,3H,5H)-trione* (**3a**) According to the general procedure, **3a** was prepared from 1,3-dimethylbarbituric acid (**1a**) and (*E*)-(2-nitrovinyl)benzene (**2a**) as white crystals (453 mg, 1.48 mmol, 99%); m.p. 85 °C; IR (KBr) ν/cm^−1^ 1738, 1671, 1552, 1372, 1228; ^1^H-NMR (CDCl_3_) *δ* 7.29 (m, 3H, Ph), 7.03 (m, 2H, Ph), 5.28 (dd, 1H, *J* = 13.9, 5.8 Hz, CH_2_NO_2_), 5.02 (dd, 1H, *J* 13.9, 5.8, CH_2_NO_2_), 4.50 (m, 1H, CHPh), 3.84 (d, 1H, *J* = 3.6 Hz, COCHCO), 3.12(s, 3H, CH_3_), 3.07(s, 3H, CH_3_); ^13^C-NMR (CDCl_3_) *δ* 166.8, 150.5, 133.7, 129.4, 127.4, 76.5, 51.5, 45.5, 28.5, 28.3; LC/MS (ESI) *m/z* 305 [M]^+^; Anal. for C_14_H_15_N_3_O_5_; calcd: C, 55.08; H, 4.95; N, 13.76; Found: C, 55.10; H, 4.95; N, 13.75. 

The structure of **3a** was confirmed by X-ray crystal structure analysis (Bruker AXS GmbH, Karlsruhe, Germany). CCDC-933479 contains the supplementary crystallographic data for this compound. These data can be obtained free of charge from the Cambridge Crystallographic Data Centre via www.ccdc.cam.ac.uk/data_request/cif. A colorless cubic crystal of the compound suitable for X-ray analysis was formed in CHCl_3_/Et_2_O at room temperature after 2 days.

*1,3-Dimethyl-5-(2-nitro-1-(p-tolyl)ethyl)pyrimidine-2,4,6(1H,3H,5H)-trione* (**3b**) According to the general procedure, **3b** was prepared from 1,3-dimethylbarbituric acid (**1a**) and (*E*)-1-methyl-4-(2-nitrovinyl)benzene (**2b**) as an oily product (460 mg, 1.44 mmol, 96%). IR (KBr) ν/cm^−1^ 1738, 1671, 1552, 1372, 1228; ^1^H-NMR (CDCl_3_) *δ* 7.08 (d, 2H, *J* = 8.0 Hz, Ph), 6.91 (d, 2H, *J* = 8.0 Hz, Ph), 5.25 (dd, 1H, *J* = 13.9, 5.8 Hz, CH_2_NO_2_), 4.99 (dd, 1H, *J* = 13.9, 5.8 Hz, CH_2_NO_2_), 4.45 (m, 1H, CHPh), 3.82 (d, 1H, *J* = 3.6 Hz, COCHCO), 3.13 (s, 3H, CH_3_), 3.08 (s, 3H, CH_3_), 2.28 (s, 3H, CH_3_); ^13^C-NMR (CDCl_3_) *δ* 166.9, 166.9, 150.6, 139.3, 130.5, 129.9, 127.3, 51.5, 45.3, 28.5, 28.3, 21.1; LC/MS (ESI): *m/z* 319 [M]^+^_;_ Anal. for C_15_H_17_N_3_O_5_; calcd: C, 56.42; H, 5.37; N, 13.16; Found: C, 56.41; H, 5.36; N, 13.17.

*5-(1-(4-Bromophenyl)-2-nitroethyl)-1,3-dimethylpyrimidine-2,4,6(1H,3H,5H)-trione* (**3c**) According to the general procedure, **3c** was prepared from 1,3-dimethylbarbituric acid (**1a**) and (*E*)-1-bromo-4-(2-nitrovinyl) benzene (**2c**) as a yellow powder (530 mg, 1.38 mmol, 92%); m.p.: 99 °C; IR (KBr) ν/cm^−1^1738, 1668, 1552, 1375; ^1^H-NMR (DMSO-*d_6_*) *δ* 7.51 (d, 2H, *J* = 7.3 Hz, Ph), 7.07 (d, 2H, *J* = 7.3 Hz, Ph), 5.40 (dd, 1H, *J* = 13.9, 5.8 Hz, CH_2_NO_2_), 5.23 (dd, 1H, *J* = 13.9, 5.8 Hz, CH_2_NO_2_), 4.29 (bs, 1H, CHPh), 4.17 (bs, 1H, COCHCO), 3.06 (s, 3H, CH_3_), 2.96 (s, 3H, CH_3_); ^13^C-NMR (DMSO-*d_6_*) *δ* 167.8, 167.5, 166.4, 151.3, 135.0, 132.2, 132.1, 131.8, 130.3, 122.1, 76.6, 52.1, 44.0, 28.5, 28.3; LC/MS (ESI) *m/z* 384 [M]^+^_;_ Anal. for C_14_H_14_BrN_3_O_5_; calcd: C, 43.77; H, 3.67; Br, 20.80; N, 10.94;Found: C, 43.79; H, 3.65; Br, 20.81; N, 10.94.

*5-(1-(4-Chlorophenyl)-2-nitroethyl)-1,3-dimethylpyrimidine-2,4,6(1H,3H,5H)-trione* (**3d**) According to the general procedure, **3d** was prepared from 1,3-dimethylbarbituric acid (**1a**) and (*E*)-1-chloro-4-(2-nitrovinyl)benzene (**2d**) as an oily product (462 mg, 1.36 mmol, 91%). IR (KBr) ν/cm^−1^ 1738, 1669, 1552, 1423, 1238; ^1^H-NMR (DMSO-*d_6_*) *δ* 7.44 (d, 2H, *J* = 8.0 Hz, Ph), 7.13 (d, 2H, *J* = 8.0 Hz, Ph), 5.41 (dd, 1H, *J* = 13.9, 5.8 Hz, CH_2_NO_2_), 5.23 (dd, 1H, *J* = 13.9, 5.8 Hz, CH_2_NO_2_), 4.30 (bs, 1H, CHPh), 4.16 (bs, 1H, COCHCO), 3.06 (s, 3H, CH_3_), 2.95 (s, 3H, CH_3_); ^13^C-NMR (DMSO-*d_6_*) *δ* 167.8, 167.5, 166.4, 151.3, 135.0, 132.2, 132.1, 131.8, 130.3, 122.1, 76.6, 52.1, 44.0, 28.5, 28.3; LC/MS (ESI) *m/z* 339 [M]^+^; Anal. for C_14_H_14_ClN_3_O_5_; calcd: C, 49.49; H, 4.15; Cl, 10.44; N, 12.37;Found: C, 49.50; H, 4.14; Cl, 10.43; N, 12.36.

*5-(1-(2,4-Dichlorophenyl)-2-nitroethyl)-1,3-dimethylpyrimidine-2,4,6(1H,3H,5H)-trione* (**3e**) According to the general procedure, **3e** was prepared from 1,3-dimethylbarbituric acid (**1a**) and (*E*)-2,4-dichloro-1-(2-nitrovinyl) benzene (**2e**) as a yellow powder (505 mg, 1.35 mmol, 90%); m.p.: 119 °C; IR (KBr) ν/cm^−1^ 1738, 1667, 1551, 1423, 1221;^1^H-NMR (DMSO-*d_6_*) *δ* 7.65 (s, 1H, Ph), 7.52 (d, 1H, *J* = 8.0 Hz, Ph), 7.45(d, 1H, *J* = 8.0 Hz, Ph), 5.31–5.22 (m, 3H, CH_2_NO_2_ and COCHCO), 4.83 (bs, 1H, CHPh); ^13^C-NMR (DMSO-*d_6_*): *δ* 167.3, 166.4, 151.7, 135.0, 130.9, 129.6, 127.9, 75.7, 51.6, 28.8, 28.7; LC/MS (ESI) *m/z* 374 [M]^+^; Anal. for C_14_H_13_Cl_2_N_3_O_5_; calcd: C, 44.94; H, 3.50; Cl, 18.95; N, 11.23; Found: C, 44.92; H, 3.51; Cl, 18.90; N, 11.25.

*5-(1-(2,6-Dichlorophenyl)-2-nitroethyl)-1,3-dimethylpyrimidine-2,4,6(1H,3H,5H)-trione* (**3f**) According to the general procedure, **3f** was prepared from 1,3-dimethylbarbituric acid (**1a**) and (*E*)-2,6-dichloro-1-(2-nitrovinyl)benzene (**2f**) as a white powder (510 mg, 1.36 mmol, 91%); m.p.: 140 °C; IR (KBr) ν/cm^−1^ 1738, 1670, 1551, 1423, 1228; ^1^H-NMR (DMSO-*d_6_*) *δ* 7.50 (d, 2H, *J* = 8.0 Hz, Ph), 7.36 (t, 1H, *J* = 8.0 Hz, Ph), 5.32 (d, 2H, *J* = 6.6 Hz, CH_2_NO_2_), 5.00 (m, 1H, CHPh), 4.11 (d, 1H, *J* = 11.0 Hz, COCHCO), 3.08 (s, 3H, CH_3_), 3.03 (s, 3H, CH_3_); ^13^C-NMR (DMSO-*d_6_*) *δ* 167.7, 166.8, 152.0, 134.7, 132.5, 131.2, 130.6, 129.3, 75.6, 50.47, 28.8, 28.7; LC/MS (ESI) *m/z* 374[M]^+^; Anal. for C_14_H_13_Cl_2_N_3_O_5_; calcd: C, 44.94; H, 3.50; Cl, 18.95; N, 11.23;Found: C, 44.92; H, 3.51; Cl, 18.90; N, 11.25.

*5-(1-(4-Methoxyphenyl)-2-nitroethyl)-1,3-dimethylpyrimidine-2,4,6(1H,3H,5H)-trione* (**3g**) According to the general procedure, **3g** was prepared from 1,3-dimethylbarbituric acid (**1a**) and (*E*)-1-methoxy-4-(2-nitrovinyl) benzene (**2g**) as an oily product (447 mg, 1.33 mmol, 89%). IR (KBr) ν/cm^−1^ 1738, 1670, 1551, 1423, 1228; ^1^H-NMR (DMSO-*d_6_*) *δ* 6.96 (d, 2H, *J* = 8.0 Hz, Ph), 6.85 (d, 2H, *J* = 8.0 Hz, Ph), 5.41 (dd, 1H, *J* = 13.9, 5.8 Hz, CH_2_NO_2_), 5.16 (dd, 1H, *J* = 13.9, 5.8 Hz, CH_2_NO_2_), 4.21 (m, 1H, CHPh), 4.04 (d, 1H, *J* = 2.9 Hz, COCHCO), 3.69 (s, 3H, CH_3_), 2.99 (s, 3H, CH_3_), 2.93 (s, 3H, CH_3_); ^13^C-NMR (DMSO-*d_6_*) *δ* 168.2, 167.7, 159.6, 151.3, 129.1, 126.6, 114.6, 77.0, 55.6,52.4, 44.7,28.4, 28.3 ; LC/MS (ESI) *m/z* 335[M]^+^; Anal. for C_15_H_17_N_3_O_6_; calcd: C, 53.73; H, 5.11; N, 12.53; Found: C, 53.74; H, 5.10; N, 12.52.

*1,3-Dimethyl-5-(2-nitro-1-(4-nitrophenyl)ethyl)pyrimidine-2,4,6(1H,3H,5H)-trione* (**3h**) According to the general procedure, **3h** was prepared from 1,3-dimethylbarbituric acid (**1a**) and (*E*)-1-nitro-4-(2-nitrovinyl)benzene (**2h**) as a dark brown powder (462 mg, 1.32 mmol, 88%); m.p.: 170 °C; IR (KBr) ν/cm^−1^ 1738, 1646, 1551, 1366, 1217; ^1^H-NMR (DMSO-*d_6_*) *δ* 7.50 (d, 2H, *J* = 8.0 Hz, Ph), 7.38 (d, 2H, *J* = 8.0 Hz, Ph), 5.32 (d, 2H, *J* = 6.6 Hz, CH_2_NO_2_), 5.00 (m, 1H, CHPh), 4.11 (d, 1H, *J* = 11 Hz, COCHCO), 3.08 (s, 3H, CH_3_), 3.03 (s, 3H, CH_3_); ^13^C-NMR (DMSO-*d_6_*) *δ* 167.7, 166.8, 152.0, 136.7, 134.7, 132.5, 131.2, 130.6, 129.3, 75.6, 50.4, 28.8, 28.7; LC/MS (ESI) *m/z* 350[M]^+^; Anal. for C_14_H_14_N_4_O_7_; calcd: C, 48.00; H, 4.03; N, 15.99; Found: C, 48.01; H, 4.01; N, 16.01.

*1,3-Dimethyl-5-(2-ferrocenyl)ethyl)pyrimidine-2,4,6(1H,3H,5H)-trione* (**3i**) According to the general procedure, **3i** was prepared from 1,3-dimethylbarbituric acid (**1a**) and (*E*)-1-ferrocenyl-2-nitroethene (**2i**) as a dark purple powder (595 mg, 1.39 mmol, 93%); m.p.: 139 °C; IR (KBr) ν/cm^−1^ 1738, 1671, 1551, 1423, 1367, 1228; ^1^H-NMR (DMSO-*d_6_*) *δ* 5.17 (d, 2H, *J* = 7.3 Hz, CH_2_NO_2_), 4.21 (bs, 5H, ferrocene), 4.00 (m, 1H, CH-ferrocene), 3.92 (d, 1H, *J* = 2.9 Hz, COCHCO), 3.38 (bs, 4H, ferrocene), 3.03 (s, 3H, CH_3_), 2.88 (s, 3H, CH_3_), 2.93 (s, 3H, CH_3_); ^13^C-NMR (DMSO-*d_6_*) *δ* 168.4, 167.6, 151.4, 83.2, 76.7, 69.4, 69.3, 68.7, 68.3, 67.7, 66.0, 52.2, 28.5, 28.2 ; LC/MS (ESI) *m/z* 427[M]^+^; Anal. for C_19_H_21_FeN_3_O_5_; calcd: C, 53.41; H, 4.95; N, 9.84; Found: C, 53.42; H, 4.94; N, 9.85.

*1,3-Dimethyl-5-(1-nitropropan-2-yl)pyrimidine-2,4,6(1H,3H,5H)-trione* (**3j**) According to the general procedure, **3j** was prepared from 1,3-dimethylbarbituric acid (**1a**) and (*E*)-1-nitroprop-1-ene (**2j**) as a yellowish oily product (350 mg, 1.44 mmol, 96%). IR (KBr) ν/cm^−1^ 1745, 1665, 1325, 1225; ^1^H-NMR (DMSO-*d_6_*) *δ* 4.93 (dd, H, *J* = 11.2, 5.2 Hz, CH_2_NO_2_), 4.70 (dd, H, *J* = 11.2, 5.2 Hz, CH_2_NO_2_), 3.98 (d, 1H, *J* = 2.4, COCHCO), 3.23 (m, 1H, CH), 3.20 (s, 3H, CH_3_), 3.02 (s, 3H, CH_3_), 0.95 (d, 3H, *J* = 5.6, CH_3_); ^13^C-NMR (DMSO-*d_6_*) *δ* 167.4, 167.3, 165.8, 151.4, 78.4, 50.3, 28.2, 27.7, 13.6; LC/MS (ESI) *m/z* 244[M]^+^; Anal. for C_9_H_13_N_3_O_5_; calcd: C, 44.44; H, 5.39; N, 17.28; Found: C, 44.46; H, 5.40; N, 17.31.

*1,3-Dimethyl-5-(2-nitro-1-(thiophen-2-yl)ethyl)pyrimidine-2,4,6(1H,3H,5H)-trione* (**3k**) According to the general procedure, **3k** was prepared from 1,3-dimethylbarbituric acid (**1a**) and (*E*)-2-(2-nitrovinyl)thiophene (**2k**) as a brown semisolid product (443 mg, 1.42 mmol, 95%). IR (KBr) ν/cm^−1^1767, 1680, 1558, 1343, 1225;^1^H-NMR (DMSO-*d_6_*) *δ* 7.45 (brs, 1H, thiophene), 6.95 (brs, 1H, thiophene), 6.89 (brs, 1H, thiophene), 5.45 (d, 1H, *J* = 10.8 Hz, CH_2_NO_2_), 5.20 (dd, 1H, *J* = 16.8, 8.8 Hz, CH_2_NO_2_), 4.66 (brs, 1H, COCHCO), 4.26 (brs, 1H, CHCH_2_NO_2_), 3.12 (s, 3H, CH_3_), 3.05 (s, 3H, CH_3_); ^13^C-NMR (DMSO-*d_6_*) *δ* 169.5, 168.7, 137.9, 135.9, 134.8, 129.8, 79.5, 29.1, 28.5, 27.5; LC/MS (ESI) *m/z* 312[M]^+^; Anal. for C_12_H_13_N_3_O_5_S; calcd: C, 46.30; H, 4.21; N, 13.50; Found: C, 46.32; H, 4.22; N, 13.48.

*6-Hydroxy-5-(2-nitro-1-phenylethyl)pyrimidine-2,4(1H,3H)-dione* (**3l**) According to the general procedure, **3l** was prepared from barbituric acid (**1b**) and (*E*)-(2-nitrovinyl)benzene (**2a**) as a white powder (401 mg, 1.45 mmol, 97%); m.p.: 165 °C; IR (KBr) ν/cm^−1^ 3204, 3015, 2920, 1740, 1550, 1363, 1217;^1^H-NMR (DMSO-*d_6_*) *δ* 11.31 (s, 2H, NH), 7.40 (m, 3H, Ph), 7.13 (m, 2H, Ph), 5.44 (dd, 1H, *J* = 13.9, 5.8 Hz, CH_2_NO_2_), 5.28 (dd, 1H, *J* = 13.9, 5.8 Hz, CH_2_NO_2_), 4.33 (m, 1H, CHPh); ^13^C-NMR (DMSO-*d_6_*) *δ* 169.7, 169.5, 150.7, 135.8, 129.3, 128.8, 128.3, 79.7, 77.4, 51.3, 43.6; LC/MS (ESI) *m/z* 277 [M]^+^; Anal. for C_12_H_11_N_3_O_5_; calcd: C, 51.99; H, 4.00; N, 15.16; Found: C, 52.01; H, 4.02; N, 15.15.

*6-Hydroxy-5-(2-nitro-1-(p-tolyl)ethyl)pyrimidine-2,4(1H,3H)-dione compound with* *diethylamine (1:1)* (**3m**) According to the general procedure, **3m** was prepared from barbituric acid (**1b**) and (*E*)-1-methyl-4-(2-nitrovinyl)benzene (**2b**) as a white powder (513 mg, 1.41 mmol, 94%); m.p.: 210 °C; IR (KBr) ν/cm^−1^ 3210, 3015, 1738, 1686, 1574, 1374; ^1^H-NMR (DMSO-*d_6_*) *δ* 9.22 (s, 2H, NH), 7.27 (d, 2H, *J* = 8.0 Hz, Ph), 6.99 (d, 2H, *J* = 8.0 Hz, Ph), 5.35 (dd, 1H, *J* = 13.9, 5.8 Hz, CH_2_NO_2_), 5.09 (dd, 1H, *J* = 13.9, 5.8 Hz, CH_2_NO_2_), 4.74(m, 1H, CHPh), 2.92 (m, 1H and 4H, COCHCO and CH_3_CH_2_NHCH_2_CH_3_), 2.21 (s, 3H, CH_3_), 1.14 (t, 6H, *J* = 7.3 Hz, CH_3_CH_2_NHCH_2_CH_3_); ^13^C-NMR (DMSO-*d_6_*) *δ* 164.7, 152.5, 141.2, 135.0, 128.7, 128.1, 85.0, 79.5, 41.9, 21.1, 11.5; LC/MS (ESI) *m/z* 364[M]^+^; Anal. for C_15_H_17_N_3_O_5_; calcd: C, 56.03; H, 6.64; N, 15.38; Found: C, 56.05; H, 6.65; N, 15.39.

*5-(1-(4-Bromophenyl)-2-nitroethyl)-6-hydroxypyrimidine-2,4(1H,3H)-dione* (**3n**) According to the general procedure, **3n** was prepared from barbituric acid (**1b**) and (*E*)-1-bromo-4-(2-nitrovinyl) benzene (**2c**) as a yellow powder (566 mg, 1.32 mmol, 88%); m.p.: 130 °C; IR (KBr) ν/cm^−1^: 3204, 3015, 2920, 1740, 1550, 1363, 1217; ^1^H-NMR (DMSO-*d_6_*) *δ* 9.22 (s, 2H, NH), 7.42 (d, 2H, *J* = 8.0 Hz, Ph), 7.18 (d, 2H, *J* = 8.0 Hz, Ph), 5.42 (dd, 1H, *J* = 13.9, 5.8 Hz, CH_2_NO_2_), 5.27 (m, 2H, CH_2_NO_2_ and CHPh); ^13^C-NMR (DMSO-*d_6_*) *δ* 169.5, 169.3, 150.7, 135.1, 133.4, 130.3, 129.3, 79.7, 77.1, 51.3, 33.2; LC/MS (ESI) *m/z* 356 [M]^+^; Anal. for C_12_H_10_BrN_3_O_5_; calcd: C, 40.47; H, 2.83; Br, 22.44; N, 11.80; Found: C, 40.50; H, 2.85; Br, 22.45; N, 11.83.

*5-(1-(4-Chlorophenyl)-2-nitroethyl)-6-hydroxypyrimidine-2,4(1H,3H)-dione* (**3o**) According to the general procedure, **3o** was prepared from barbituric acid (**1b**) and (*E*)-1-chloro-4-(2-nitrovinyl) benzene (**2d**) as a white powder (512 mg, 1.33 mmol, 89%); m.p.: 80 °C; IR (KBr) ν/cm^−1^ 3208, 3018, 2980, 1740, 1699, 1555, 1363, 1217; ^1^H-NMR (DMSO-*d_6_*) *δ* 9.22 (s, 2H, NH), 7.55 (m, 4Ph), 5.37 (dd, 1H, *J* = 13.9 Hz, CH_2_NO_2_), 5.07 (dd, 1H, *J* = 13.9 Hz, CH_2_NO_2_), 4.73 (m, 1H, CHPh); ^13^C-NMR (DMSO-*d_6_*) *δ* 164.4, 152.3, 143.5, 132.2, 131.8, 131.4, 130.5, 119.2, 84.8, 78.9, 29.4; LC/MS (ESI) *m/z* 311 [M]^+^; Anal. for C_12_H_10_ClN_3_O_5_; calcd:C, 46.24; H, 3.23; Cl, 11.37; N, 13.48; Found: C, 46.21; H, 3.22; Cl, 11.40; N, 13.45.

*5-(1-(2,4-Dichlorophenyl)-2-nitroethyl)-6-hydroxypyrimidine-2,4(1H,3H)-dione compound with diethylamine (1:1)* (**3p**) According to the general procedure, **3p** was prepared from barbituric acid (**1b**) and (*E*)-2,4-dichloro-1-(2-nitrovinyl)benzene (**2e**) as a beige powder (534 mg, 1.27 mmol, 85%); m.p.: 190 °C; IR (KBr) ν/cm^−1^ 3151, 2986, 1697, 1590, 1376; ^1^H-NMR (DMSO-*d_6_*) *δ* 9.18 (s, 2H, NH), 8.30 (bs, 1H, OH), 7.83 (d, 2H, *J* = 8.8 Hz, Ph), 7.44(s, 1H, Ph), 7.30 (d, 2H, *J* = 8.8 Hz, Ph), 5.35 (dd, 1H, *J* = 13.9, 5.8 Hz, CH_2_NO_2_), 5.14 (dd, 1H, *J* = 13.9, 5.8 Hz, CH_2_NO_2_), 4.90 (m, 1H, CHPh), 2.92 (m, 1H and 4H, COCHCO and CH_3_CH_2_NHCH_2_CH_3_), 2.21 (s, 3H, CH_3_), 1.14 (t, 6H, *J* = 7.3 Hz, CH_3_CH_2_NHCH_2_CH_3_); ^13^C-NMR (DMSO-*d_6_*) *δ* 165.0, 152.5, 140.2, 134.1, 132.7, 131.7, 128.5, 127.3, 82.5, 77.3, 41.9, 21.1, 11.5; LC/MS (ESI) *m/z* 419 [M]^+^; Anal. for C_16_H_20_Cl_2_N_4_O_5_; calcd: C, 45.84; H, 4.81; Cl, 16.91; N, 13.36; Found: C, 45.87; H, 4.83; Cl, 16.90; N, 13.35.

*5-(1-(2,6-Dichlorophenyl)-2-nitroethyl)-6-hydroxypyrimidine-2,4(1H,3H)-dione* (**3q**) According to the general procedure, **3q** was prepared from barbituric acid (**1b**) and (*E*)-2,6-dichloro-1-(2-nitrovinyl) benzene (**2f**) as a yellow powder (540 mg, 1.29 mmol, 86%); m.p.: 130 °C; IR (KBr) ν/cm^−1^ 3155, 2986, 1740, 1670, 1551, 1423, 1228;^1^H-NMR (DMSO-*d_6_*) *δ* 11.38 (s, 2H, NH), 7.63 (d, 2H, *J* = 8.8 Hz, Ph), 7.46 (s, 1H, Ph), 7.36 (d, 2H, *J* = 8.8 Hz, Ph), 5.37 (dd, 1H, *J* = 13.9, 5.8 Hz, CH_2_NO_2_), 5.25 (dd, 1H, *J* = 13.9, 5.8 Hz, CH_2_NO_2_), 5.04 (m, 1H, CHPh); ^13^C-NMR (DMSO-*d_6_*) *δ* 169.6, 168.4, 151.3, 133.1, 130.9, 129.8, 129.2, 75.8, 49.2; LC/MS (ESI) *m/z* 419 [M]^+^; Anal. for C_12_H_9_Cl_2_N_3_O_5_; calcd: C, 41.64; H, 2.62; Cl, 20.49; N, 12.14; Found: C, 41.65; H, 2.61; Cl, 20.50; N, 12.13.

*6-Hydroxy-5-(1-(4-methoxyphenyl)-2-nitroethyl)pyrimidine-2,4(1H,3H)-dione compound with diethylamine (1:1)* (**3r**) According to the general procedure, **3r** was prepared from barbituric acid (**1b**) and (*E*)-4-methoxy-1-(2-nitrovinyl)benzene (**2g**) as a yellow powder (501 mg, 1.32 mmol, 88%); m.p.: 152 °C; IR (KBr) ν/cm^−1^ 3459, 3016, 2970, 1740, 1571, 1365; ^1^H-NMR (DMSO-*d_6_*) *δ* 9.07 (s, 2H, NH), 7.32 (d, 2H, *J* = 8.8 Hz, Ph), 6.75(s, 1H, Ph), 7.30 (d, 2H, *J* = 8.8 Hz, Ph), 5.31 (dd, 1H, *J* = 13.9, 5.8 Hz, CH_2_NO_2_), 5.06 (dd, 1H, *J* = 13.9, 5.8 Hz, CH_2_NO_2_), 4.68 (m, 1H, CHPh), 3.68 (s, 3H, OCH_3_), 2.93 (m, 1H and 4H, COCHCO and CH_3_CH_2_NHCH_2_CH_3_), 1.15 (t, 6H, *J* = 7.3 Hz, CH_3_CH_2_NHCH_2_CH_3_); ^13^C-NMR (DMSO-*d_6_*) *δ* 164.6, 157.9, 152.5, 136.4, 129.3, 113.5, 85.0, 79.8, 55.4, 41.9, 11.6; LC/MS (ESI) *m/z* 380[M]^+^; Anal. for C_17_H_24_N_4_O_6_; calcd: C, 53.68; H, 6.36; N, 14.73; Found: C, 53.68; H, 6.36; N, 14.73.

*5-(2-Ferrocenyl)ethyl)6-Hydroxypyrimidine-2,4(1H,3H)-dione compound with diethylamine(1:1)* (**3s**) According to the general procedure, **3s** was prepared from barbituric acid (**1b**) and (*E*)-1-ferrocenyl-2-nitroethene (**2i**) as a brown powder (550 mg, 1.38 mmol, 92%); m.p.: 190 °C; IR (KBr) ν/cm^−1^ 3449, 3016, 2970, 1738, 1546, 1365, 1217;^1^H-NMR (DMSO-*d_6_*) *δ* 8.98 (s, 2H, NH), 5.16 (t, 1H, *J* = 9.5 Hz, CH_2_NO_2_), 4.89 (dd, 1H, *J* = 13.9, 5.8 Hz, CH_2_NO_2_), 4.53(m, 1H, CHPh), 4.25-3.93 (m, 10H, ferrocene and COCHCO), 2.92 (bs, 4H, CH_3_CH_2_NHCH_2_CH_3_), 1.15 (bs, 6H, CH_3_CH_2_NHCH_2_CH_3_); ^13^C-NMR (DMSO-*d_6_*) *δ* 164.5, 152.5, 92.3, 84.7, 79.6, 69.3, 68.8, 67.8, 66.7, 66.4, 41.8, 34.7, 11.6; LC/MS (ESI) *m/z* 399 [M]^+^; Anal. for C_17_H_17_FeN_3_O_5_; calcd: C, 51.15; H, 4.29; N, 10.53; Found: C, 51.17; H, 4.30; N, 10.54.

*6-Hydroxy-5-(2-nitro-1-(4-nitrophenyl)ethyl)pyrimidine-2,4(1H,3H)-dione compound with diethylamine (1:1)* (**3t**). According to the general procedure, **3t** was prepared from barbituric acid (**1b**) and (*E*)-4-nitro-1-(2-nitrovinyl)benzene (**2h**) as a yellow powder (515 mg, 1.3 mmol, 87%); m.p.: 185 °C; IR (KBr) ν/cm^−1^ 3445, 3015, 2970, 1738, 1575, 1373, 1216;^1^H-NMR (DMSO-*d_6_*) *δ* 9.18 (s, 2H, NH), 8.13(d, 1H, *J* = 7.3 Hz, Ph), 7.70(d, 1H, *J* = 8.0 Hz, Ph), 7.56(t, 1H, *J* = 8.0 Hz, Ph), 7.36(t, 1H, *J* = 7.3 Hz, Ph), 5.57(dd, 1H, *J* = 11.7, 8.0 Hz, CH_2_NO_2_), 5.11 (t, 1H, *J* = 8.0 Hz, COCHCO), 5.06 (dd, 1H, *J* = 11.7, 8.0 Hz, CH_2_NO_2_), 3.37 (bs, 1H, CHPh), 2.90 (q, *J* = 7.3 Hz, 4H, CH_3_CH_2_NHCH_2_CH_3_), 1.13 (t, 6H, *J* = 7.3 Hz, CH_3_CH_2_NHCH_2_CH_3_); ^13^C-NMR (DMSO-*d_6_*) *δ* 164.9, 152.5, 149.5, 138.6, 133.0, 131.4, 127.6, 123.6, 84.5, 78.0, 41.9, 35.1, 11.5; LC/MS (ESI) *m/z* 395[M]^+^; Anal. for C_16_H_21_N_5_O_7_; calcd: C, 48.61; H, 5.35; N, 17.71; Found: C, 48.59; H, 5.34; N, 17.68.

## 4. Conclusions

A very convenient procedure for the syntheses of pyrimidine derivatives by Michael addition of cyclic 1,3-dicarbonyl compounds to a range of nitroalkenes using a simple NHEt_2_/H_2_O medium has been developed. The reaction scope is substantial and a number of substituted barbituric acids and nitroalkenes could be successfully applied to give multifunctional pyrimidine derivatives. These reactions gave high yields of products in short periods of time. The study of the full scope of this asymmetric transformation and its application in the synthesis of biologically active molecules are currently underway in our laboratory.

## Supplementary Materials

Supplementary materials can be accessed at: http://www.mdpi.com/1420-3049/19/1/1150/s1.

## References

[1] Sibi M.P., Manyem S. (2000). Enantioselective conjugate additions. Tetrahedron.

[2] Berner O.M., Tedeschi L., Enders D. (2002). Asymmetric Michael additions to nitroalkenes. Eur. J. Org.Chem..

[3] Christoffers J., Baro A. (2003). Construction of quaternary stereocenters: New perspectives through enantioselective Michael reactions. Angew. Chem. Int. Ed..

[4] Notz W., Tanaka F., Barbas C.F. (2004). Enamine-based organocatalysis with proline and diamines: The development of direct catalytic asymmetric Aldol, Mannich, Michael, and Diels-alder reactions. Acc. Chem. Res..

[5] Ballini R., Bosica G., Fiorini D., Palmieri A., Petrini M. (2005). Conjugate additions of nitroalkanes to electron-poor alkenes: Recent results. Chem. Rev..

[6] Sulzer-Moss S., Alexakis A. (2007). Chiral amines as organocatalysts for asymmetric conjugate addition to nitroolefins and vinyl sulfones via enamine activation. Chem. Commun..

[7] Krause N., Hoffmann-Roder A. (2001). Recent advances in catalytic enantioselective Michael additions. Synthesis.

[8] Ono N. (2001). The Nitro Group in Organic Synthesis.

[9] Czekelius C., Carreira E.M. (2005). Convenient transformation of optically active into chiral aldoximes and nitriles. Angew. Chem. Int. Ed..

[10] Calderari G., Seebach D. (1985). Asymmetrische Michael-additionen. Stereoselektive alkylierung chiraler, nicht racemischer enolate durch nitroolefine. Herstellung enantiomerenreiner γ-aminobuttersäure- und bernsteinsäure-derivate. Helv. Chim. Acta.

[11] Rosini G., Ballini R. (1988). Functionalized nitroalkanes as useful reagents for alkyl anion synthons. Synthesis.

[12] Barrett A.G.M., Graboski G. (1986). Conjugated nitroalkenes: Versatile intermediates in organic synthesis. Chem. Rev..

[13] Ballini R., Petrini M. (2004). Recent synthetic developments in the nitro to carbonyl conversion (Nef reaction). Tetrahedron.

[14] Shi M., Lei Z.Y., Zhao M.X., Shi J.W. (2007). A highly efficient asymmetric Michael addition of anthrone to nitroalkenes with cinchona organocatalysts. Tetrahedron Lett..

[15] Cai J.F., Guan Z., He Y.H. (2011). The lipase-catalyzed asymmetric C–C Michael addition. J. Mol. Catal. B Enzym..

[16] Nigmatov A.G., Kuchurov I.V., Siyutkin D.E., Zlotin S.G. (2012). Enantioselective addition of carbon acids to α-nitroalkenes: The first asymmetric aminocatalytic reaction in liquefied carbon dioxide. Tetrahedron Lett..

[17] Chen F.X., Shao C., Wang Q., Gong P., Zhang D.Y., Zhang B.Z., Wang R. (2007). An enantioselective Michael addition of malonate to nitroalkenes catalyzed by low loading demethylquinine salts in water. Tetrahedron Lett..

[18] Albrecht B., Harald G. (2005). Asymmetric Organocatalysis—From Biomimetic Concepts to Applications in Asymmetric Synthesis.

[19] Hayashi Y., Gotoh T., Hayasji T., Shoji M. (2005). Diphenylprolinol silyl ethers as efficient organocatalysts for the asymmetric Michael reaction of aldehydes and nitroalkenes. Angew. Chem. Int. Ed..

[20] Huang H.B., Jacobsen E.N. (2006). Highly enantioselective direct conjugate addition of ketones to nitroalkenes promoted by a chiral primary amine-thiourea catalyst. J. Am. Chem. Soc..

[21] Watanabe M., Ikagawa A., Wang H., Murata K., Ikariya T. (2004). Catalytic enantioselective Michael addition of 1,3-dicarbonyl compounds to nitroalkenes catalyzed by well-defined chiral ru amido complexes. J. Am. Chem. Soc..

[22] Evans D.A., Seidel D. (2005). Ni(II)-Bis[(R,R)-*N,N*′-dibenzylcyclohexane-1,2-diamine]Br2 catalyzed enantioselective Michael additions of 1,3-dicarbonyl compounds to conjugated nitroalkenes. J. Am. Chem. Soc..

[23] Lu S.F., Du D.M., Xu J., Zhang S.W. (2006). Asymmetric Michael addition of nitroalkanes to nitroalkenes catalyzed by C2-symmetric tridentate bis(oxazoline) and bis(thiazoline) zinc complexes. J. Am. Chem. Soc..

[24] Dijk E.W., Boersma A.J., Feringa B.L., Roelfes G. (2010). On the role of DNA in DNA-based catalytic enantioselective conjugate addition reactions. Org. Biomol. Chem..

[25] Izquierdo C., Luis-Barrera J., Fraile A., Alemán J. (2014). 1,4-Michael additions of cyclic-β-ketoesters catalyzed by DNA in aqueous media. Catal. Commu..

[26] Lindstrom U.M. (2002). Stereoselective organic reactions in water. Chem. Rev..

[27] Habibi A., Tarameshloo Z. (2011). A new and convenient method for synthesis of barbituric acid derivatives. J. Iran. Chem. Soc..

[28] Rastaldo R., Penna C., Pagliaro P. (2001). Comparison between the effects of pentobarbital or ketamine/nitrous oxide anesthesia on metabolic and endothelial components of coronary reactive hyperemia. Life Sci..

[29] Jursic B.S., Stevens D.E. (2003). Transition metal free reductive dimerization of nitrogen containing barbituric acid benzylidenes. J. Heterocycl. Chem..

[30] Wolff M.E. (1997). Burger’s Medicinal Chemistry and Drug Discovery.

[31] Holtkamp M., Meierkord H. (2007). Anticonvulsant, antiepileptogenic, and antiictogenic pharmacostrategies. Cell. Mol. Life Sci..

[32] Kotha S., Dep A., Kumar R. (2005). Design and synthesis of spiro-annulated barbituric acid derivatives and its analogs by ring-closing metathesis reaction as key steps. Bioorg. Med. Chem. Lett..

[33] Barakat A., Al Majid A.M.A., Shahidul Islam M., Al-Othman Z.A. (2013). Highly enantioselective Friedel−Crafts alkylations of indoles with *α,β*-unsaturated ketones under Cu(II)-simple oxazoline-imidazoline catalysts. Tetrahedron.

[34] Barakat A., Al-Majid A.M.  (2013). Synthesis, structural characterization of monodentate phosphite ligands and phosphite ruthenium complexes derived from d-mannitol. Arab. J. Chem..

[35] Al-Majid A.M.A., Barakat A., Mabkhot Y.N., Islam M.S. (2012). Synthesis and characterization of privileged monodentatephosphoramidite ligands and chiral brønsted acid derived from d-mannitol. Int. J. Mol. Sci..

[36] Al-Majid A.M., Barakat A., AL-Najjar H.J., Mabkhot Y.N., Ghabbour H.A., Fun H.K. (2013). Tandem Aldol-Michael reactions in aqueous diethylamine medium: A greener and efficient approach to bis-pyrimidine derivatives. Int. J. Mol. Sci..

[37] Barakat A., Al-Majid A.M., AL-Najjar H.J., Mabkhot Y.N., Ghabbour H.A., Fun H.K. (2014). An efficient and green procedure for synthesis of rhodanine derivatives by Aldol-thia-Michael protocol using aqueous diethylamine. RSC Adv..

[38] Abaee M.S., Cheraghi S., Navidipoor S., Mojtahedi M.M., Forghani S. (2012). An efficient tandem aldol condensation-thia-Michael addition process. Tetrahedron Lett..

[39] Gruttadauria M., Giacalone F., Marculesco A.M., Meo P.L., Riela S., Noto R. (2007). Hydrophobically directed Aldol reactions: Polystyrene-supported l-proline as a recyclable catalyst for direct asymmetric Aldol reactions in the presence of water. Eur. J. Org. Chem..

[40] Breslow R. (2004). Determining the geometries of transition states by use of antihydrophobic additives in water. Acc. Chem. Res..

[41] Blackmond D.G., Armstrong A., Coombe V., Wells A. (2007). Water in organocatalytic processes: Debunking the myths. Angew. Chem. Int. Ed. Engl..

