# Supplementary material for: A Greener, Efficient Approach to Michael Addition of Barbituric Acid to Nitroalkene in Aqueous Diethylamine Medium
Source: Molecules. 2014 Jan 17;19(1):1150–62. doi: 10.3390/molecules19011150 (PMC6271361; doi:10.3390/molecules19011150)

Supplementary Materials

Figure S1. The crystal packing of compound **3a**. Dashed line indicates the intermolecular interactions. The hydrogens do not involved in inter molecular interactions are omitted for clarity.

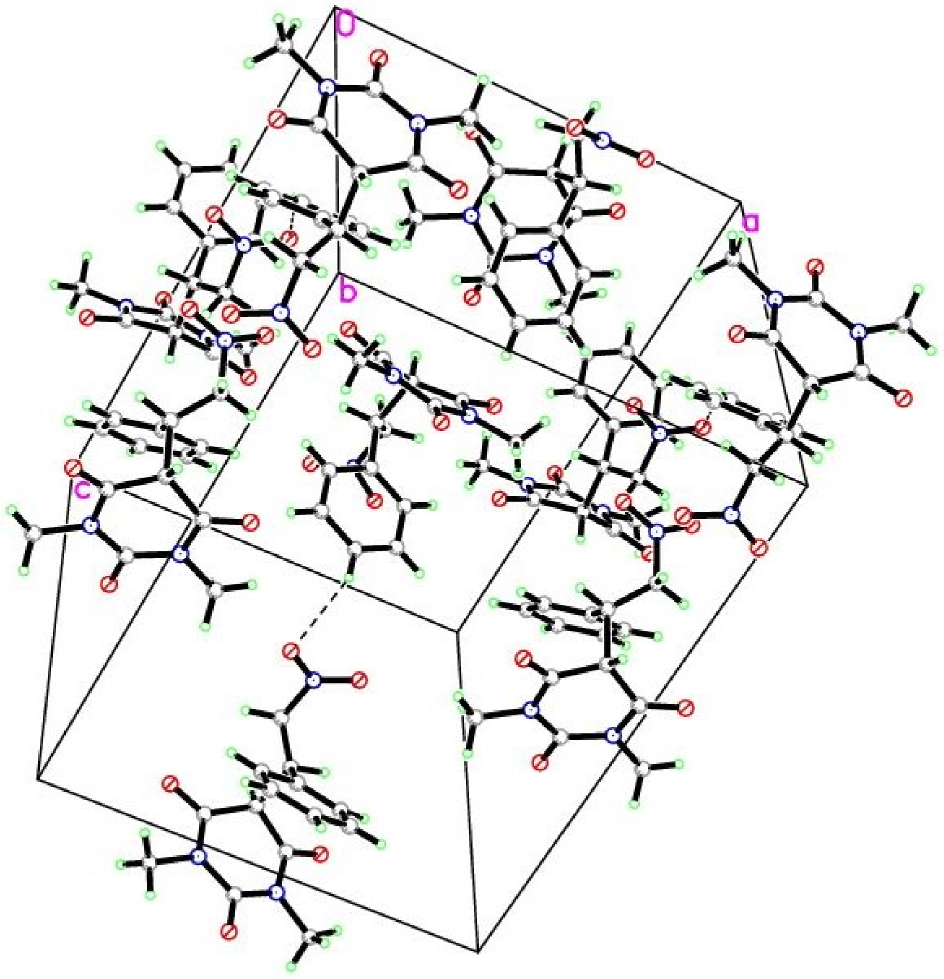

Figure S1. *Cont.*

	Crystal Data of **3a** (CCDC—933479)
Empirical formula	$C_{14} H_{20}N_3O_5$
Formula weight	305.29
Temperature	293(2) K
Wavelength (Cu *K*α radiation, λ)	1.54178 Å
Crystal system	Orthorhombic
Space group	$P2_12_12_1$
Unit cell dimensions	a = 12.5332 (2) Å α= 90.00°<br>b = 14.3843 (2) Å β= 90.00°<br>c = 16.2357 (3) Å γ= 90.00°
Volume	2926.99 (8) $Å^3$
Z	4
Density (calculated)	1.386 kg/m^3
Absorption coefficient	0.90 mm^{-1}
F(000)	1280
Crystal size	0.48 × 0.37 × 0.25 mm
Theta range for data collection	4.1 to 69.8°.
Index ranges	−14≤ h ≤15, −17 ≤ k ≤ 17, −19≤ l ≤ 12
Reflections collected/ unique	14208/ 3541 [R(int) = 0.0358]
Completeness to theta = 69.8°	99.5%
Absorption correction	multi-scan
Refinement	method Full-matrix least-squares on F^2
Goodness-of-fit on F^2	1.06
Final R indices [I > 2sigma(I)]	R1 = 0.0784, wR2 = 0.2214
R indices (all data)	R1 = 0.0819, wR2 = 0.2176
Largest diff. peak and hole	0.73 and −0.43 e $Å^{-3}$

Hydrogen-bond geometry (Å, °) of **3a**

D—H···*A*	*D*—H	H···*A*	*D*···*A*	*D*—H···*A*
C2A—H1···O1A	0.9700	2.3000	2.733 (7)	106.00
C2A—H3···O3Bi	0.9600	2.6000	3.510 (7)	159.00
C4A—H4···O2A	0.9600	2.2600	2.706 (6)	108.00
C7A—H8···O1A	0.9800	2.5900	3.036 (8)	108.00
C2B—H10···O1B	0.9700	2.2800	2.723 (7)	106.00
C11A—H11A···O5Aii	0.9300	2.5000	3.321 (7)	147.00
C4B—H13···O3B	0.9600	2.3000	2.730 (7)	106.00

Symmetry codes: (i) −*x*, *y* + 1/2, −*z* + 1/2; (ii) –*x* + 1, *y* + 1/2, −*z* − 1/2.

Figure S2. ^{1}H-NMR ($CDCl_3$) of **3a**.

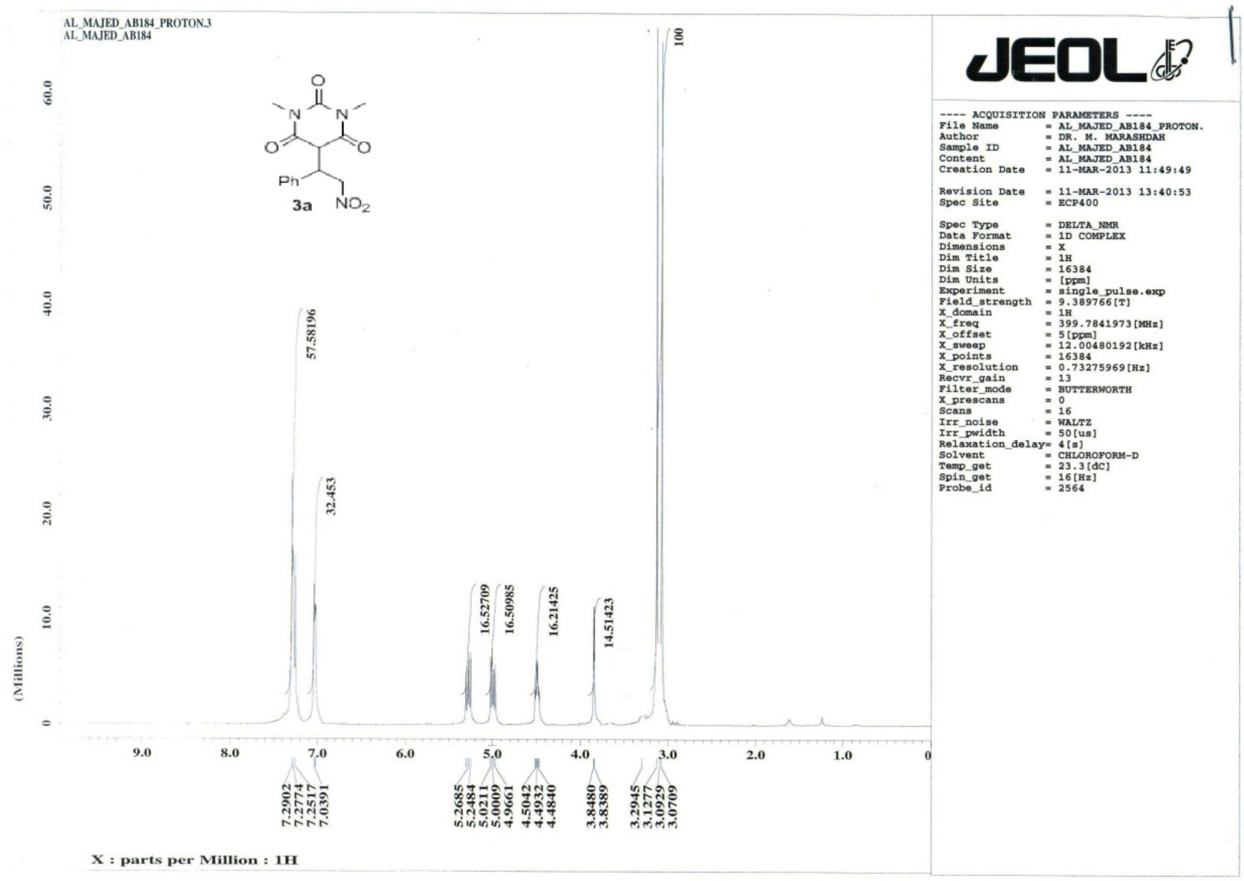

Figure S3. ^{13}C-NMR ($CDCl_3$) of **3a**.

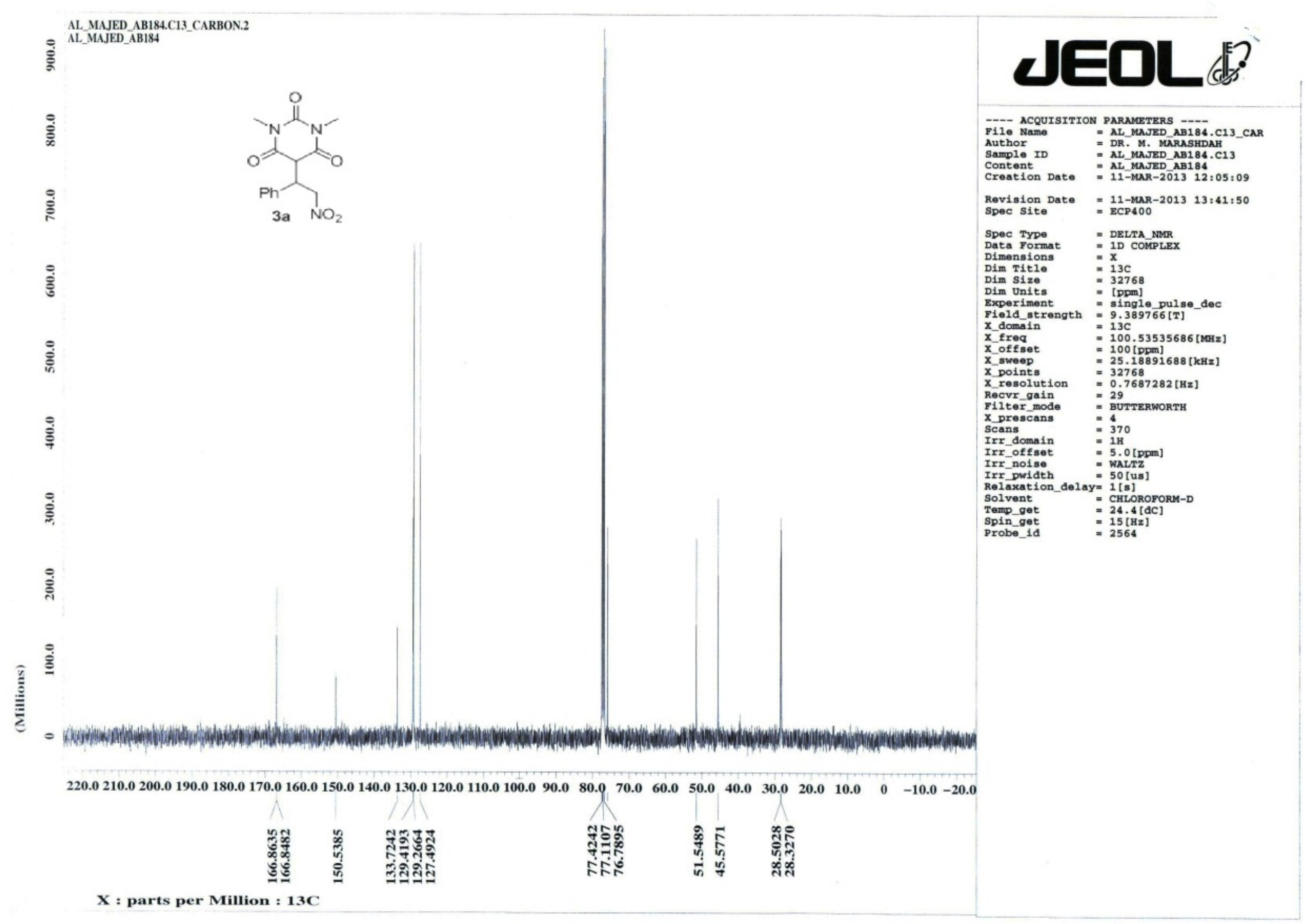

Figure S4. ^{1}H-NMR ($CDCl_3$) of **3b**.

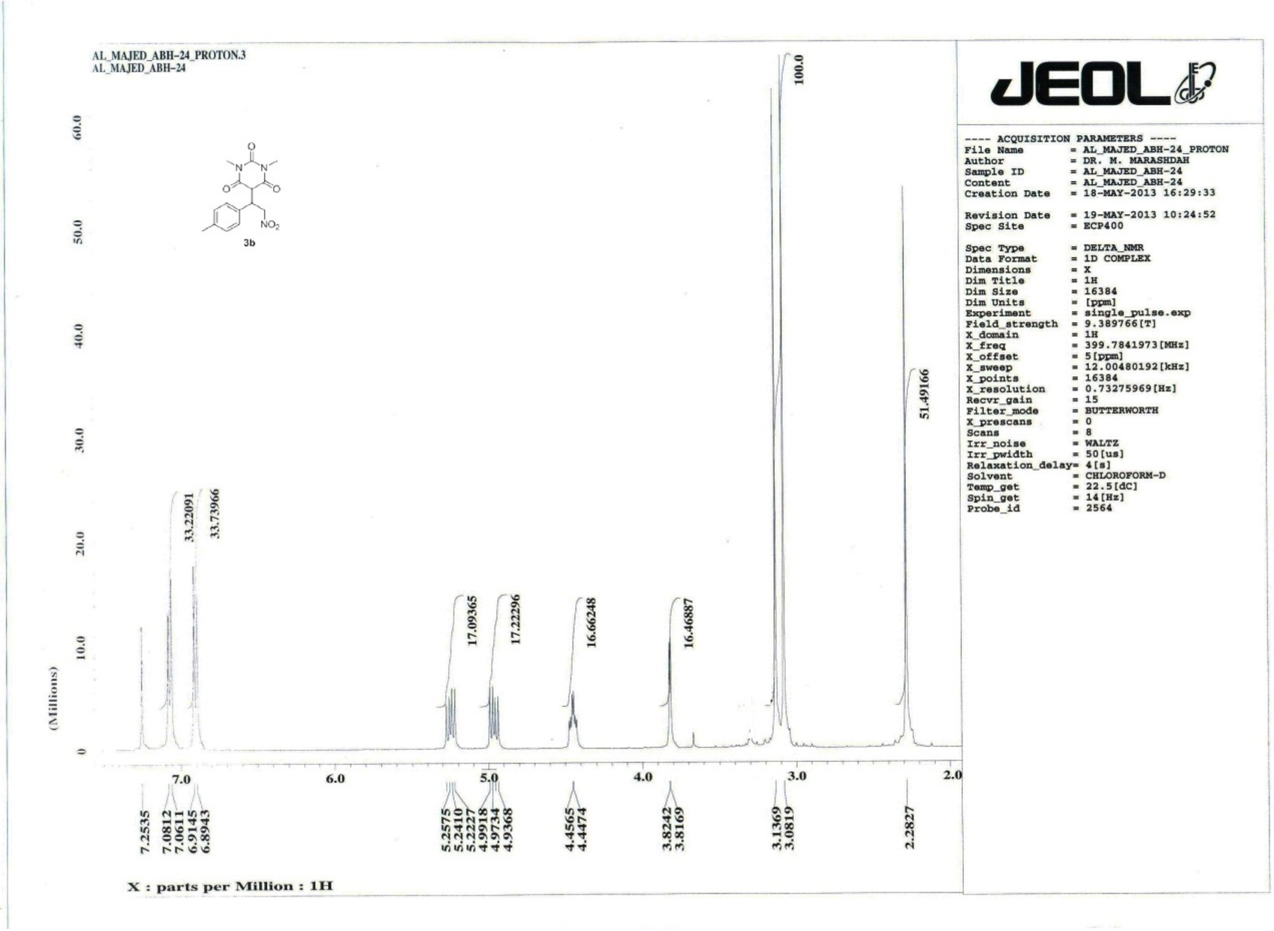

Figure S5. ^{13}C-NMR ($CDCl_3$) of **3b**.

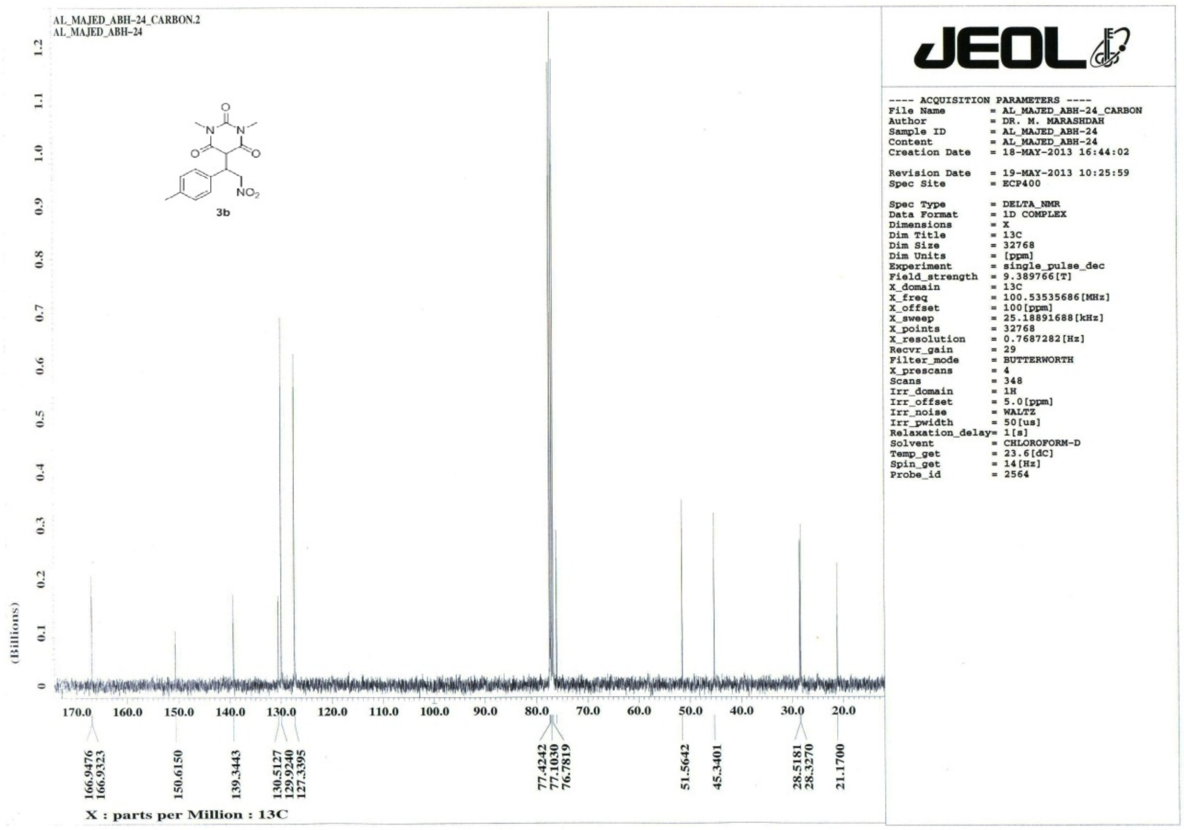

Figure S6. ^{1}H-NMR (DMSO-d_6) of **3c**.

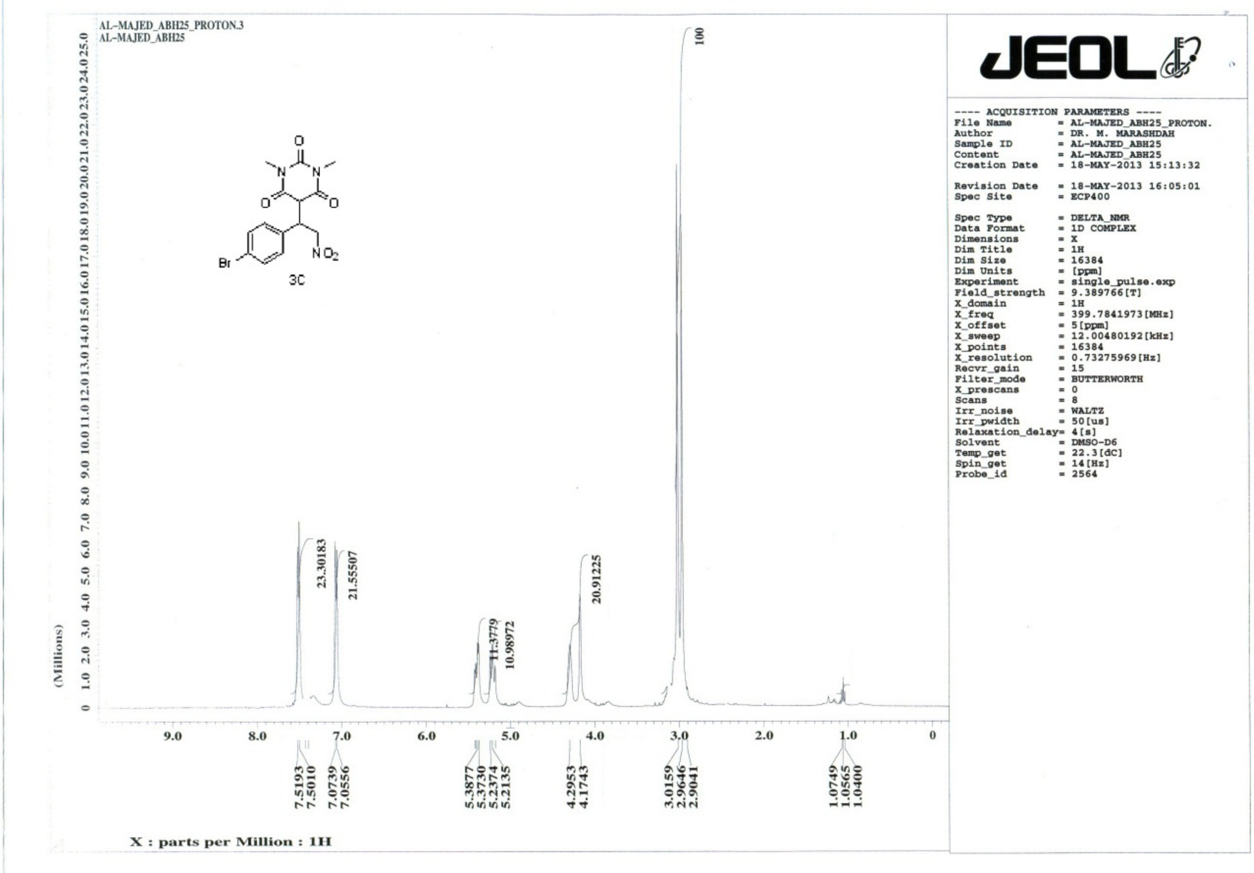

Figure S7. ^{13}C-NMR (DMSO-d_6) of **3c**.

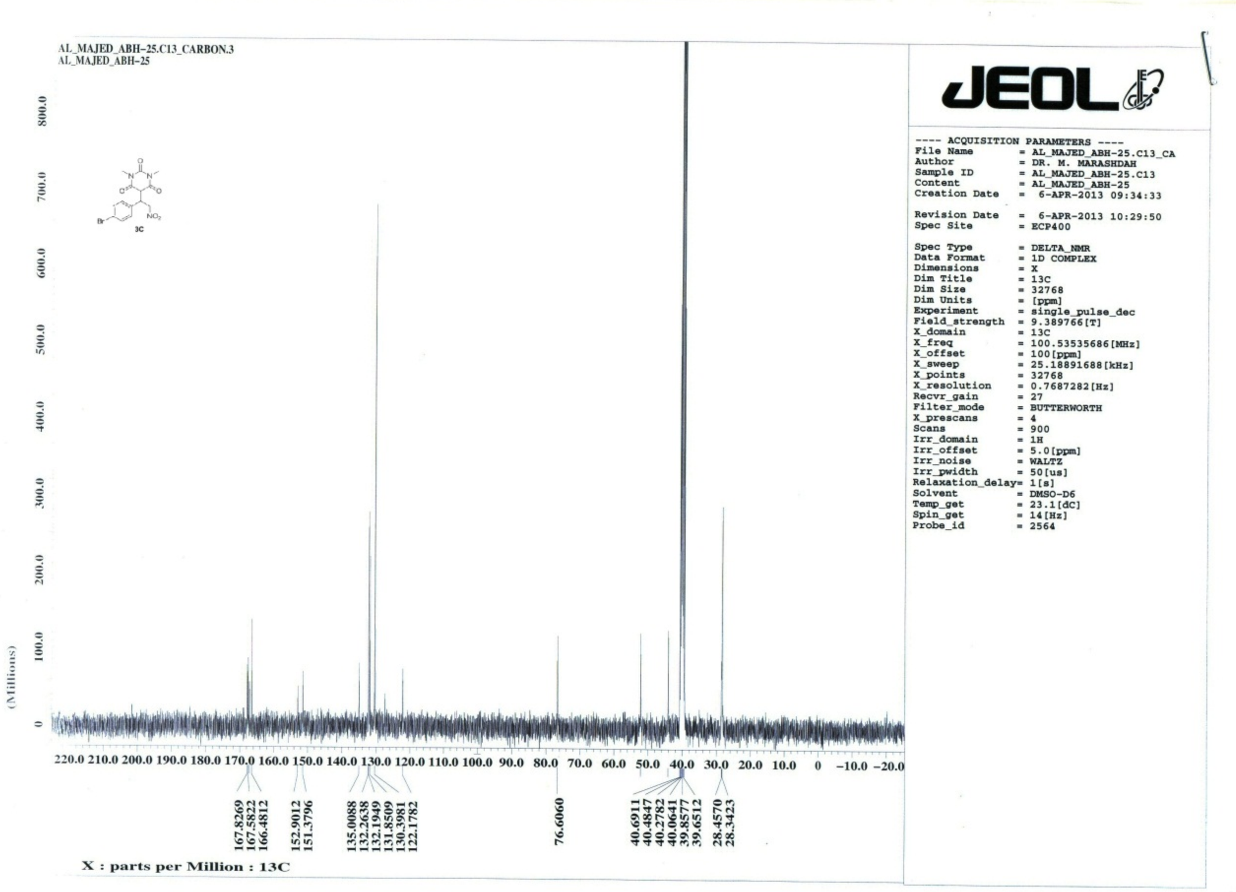

Figure S8. ^{1}H-NMR (DMSO-d_6) of **3d**.

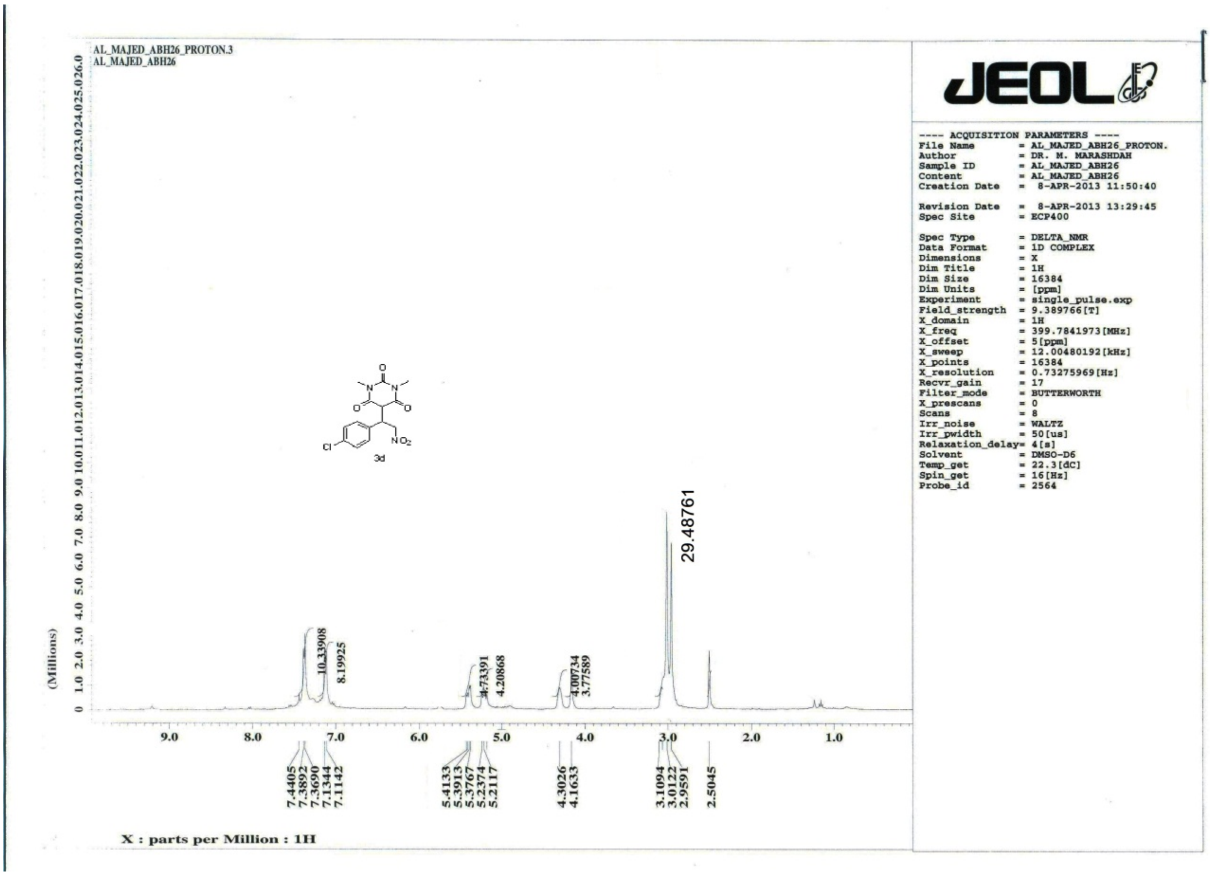

Figure S9. ^{13}C-NMR (DMSO-d_6) of **3d**.

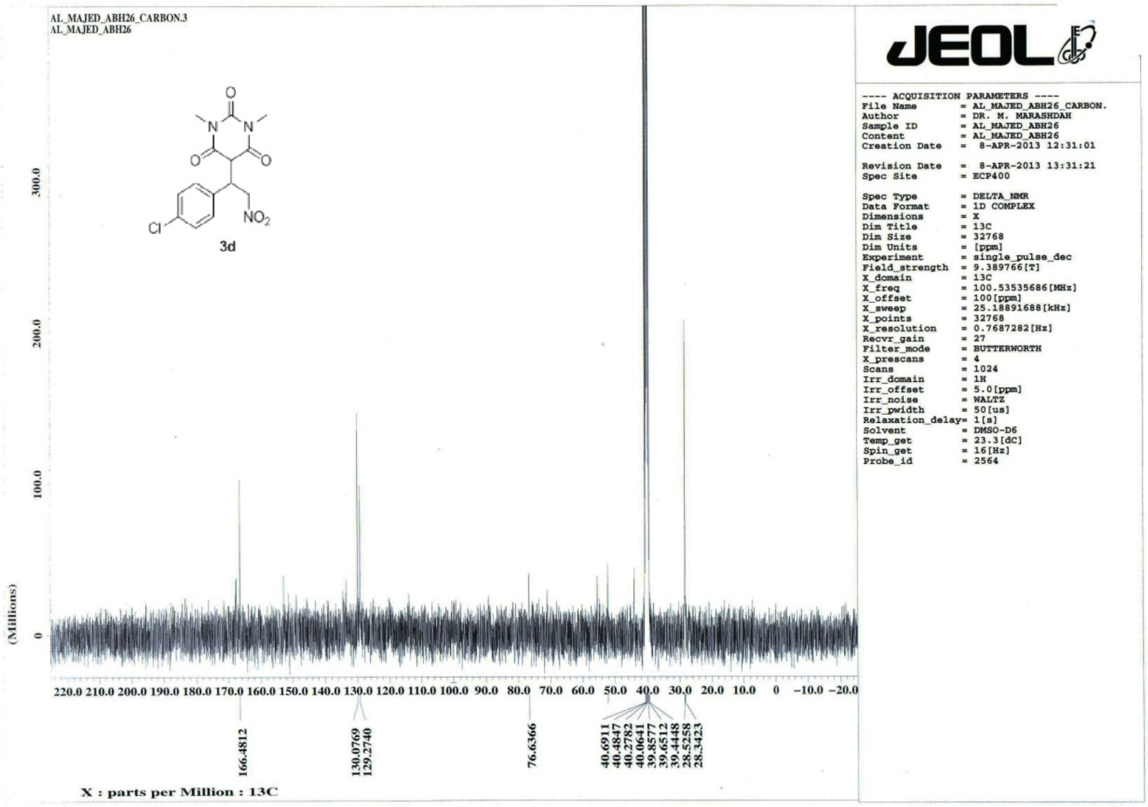

Figure S10. ^{1}H-NMR (DMSO-d_6) of **3e**.

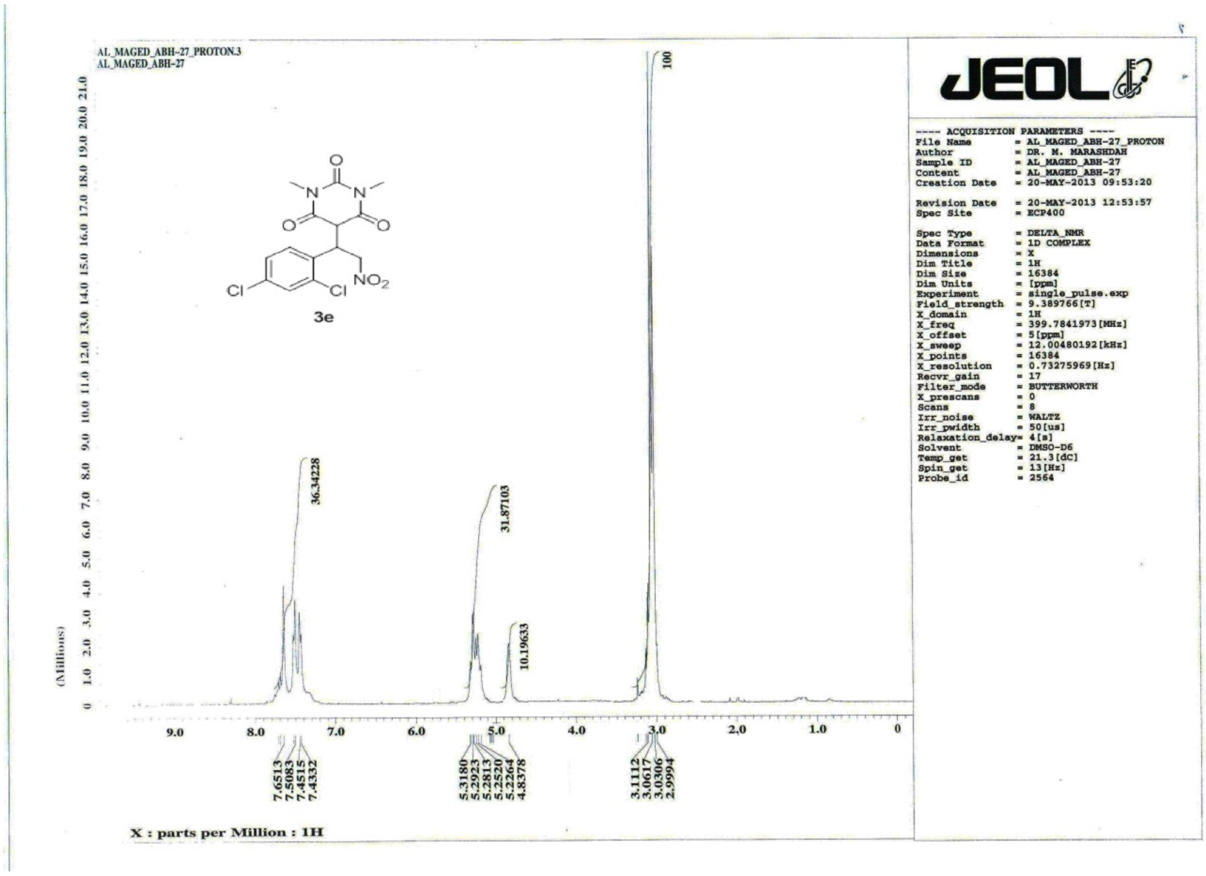

Figure S11. ^{13}C-NMR (DMSO-d_6) of **3e**.

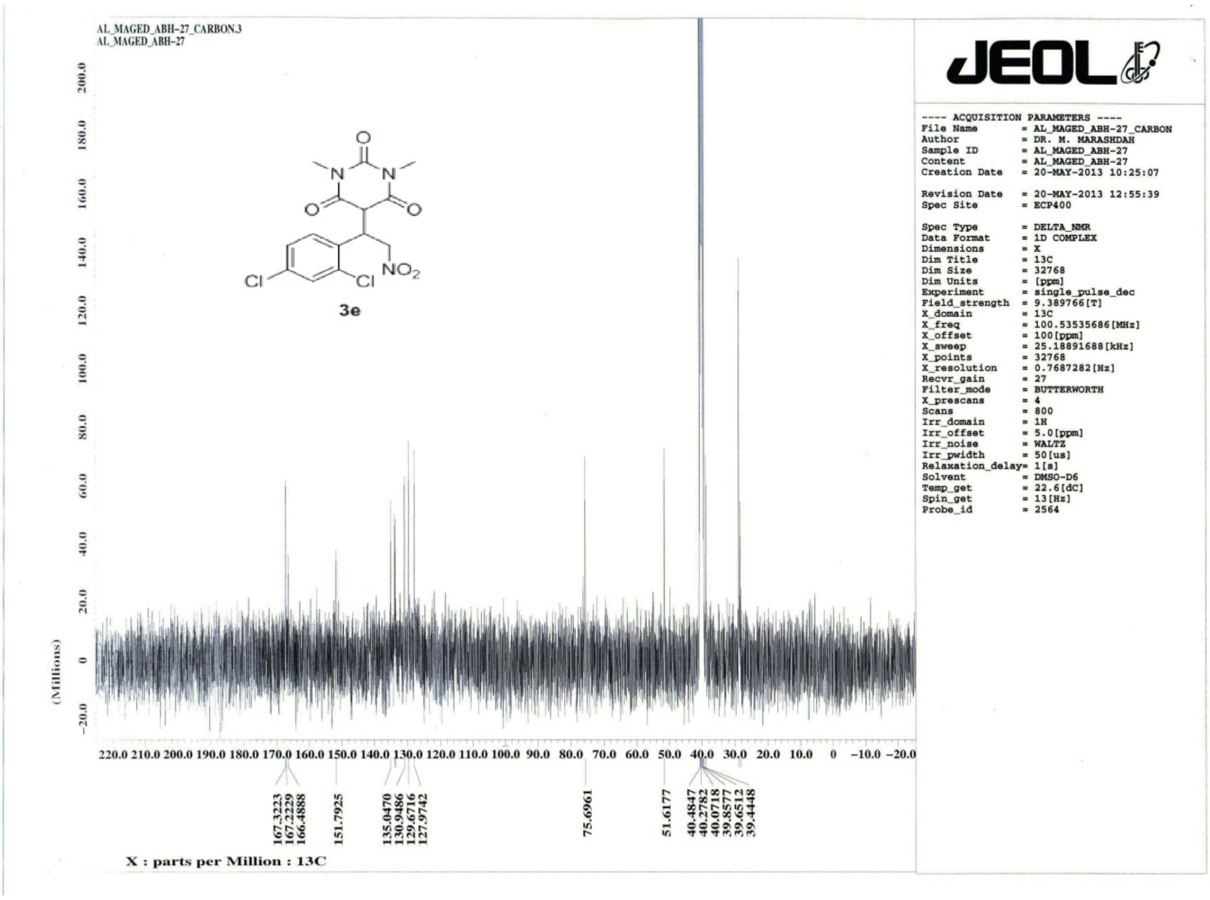

Figure S12. ^{1}H-NMR (DMSO-d_6) of **3g**.

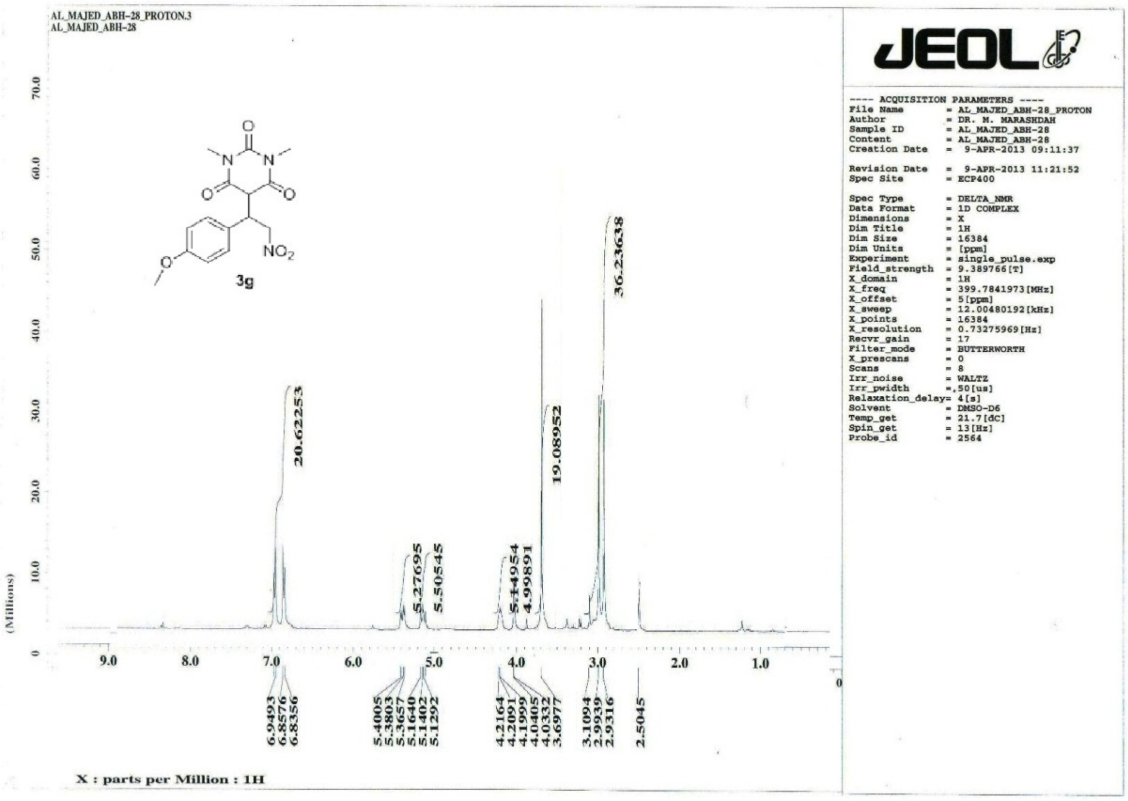

Figure S13. ^{13}C-NMR (DMSO-d_6) of **3g**.

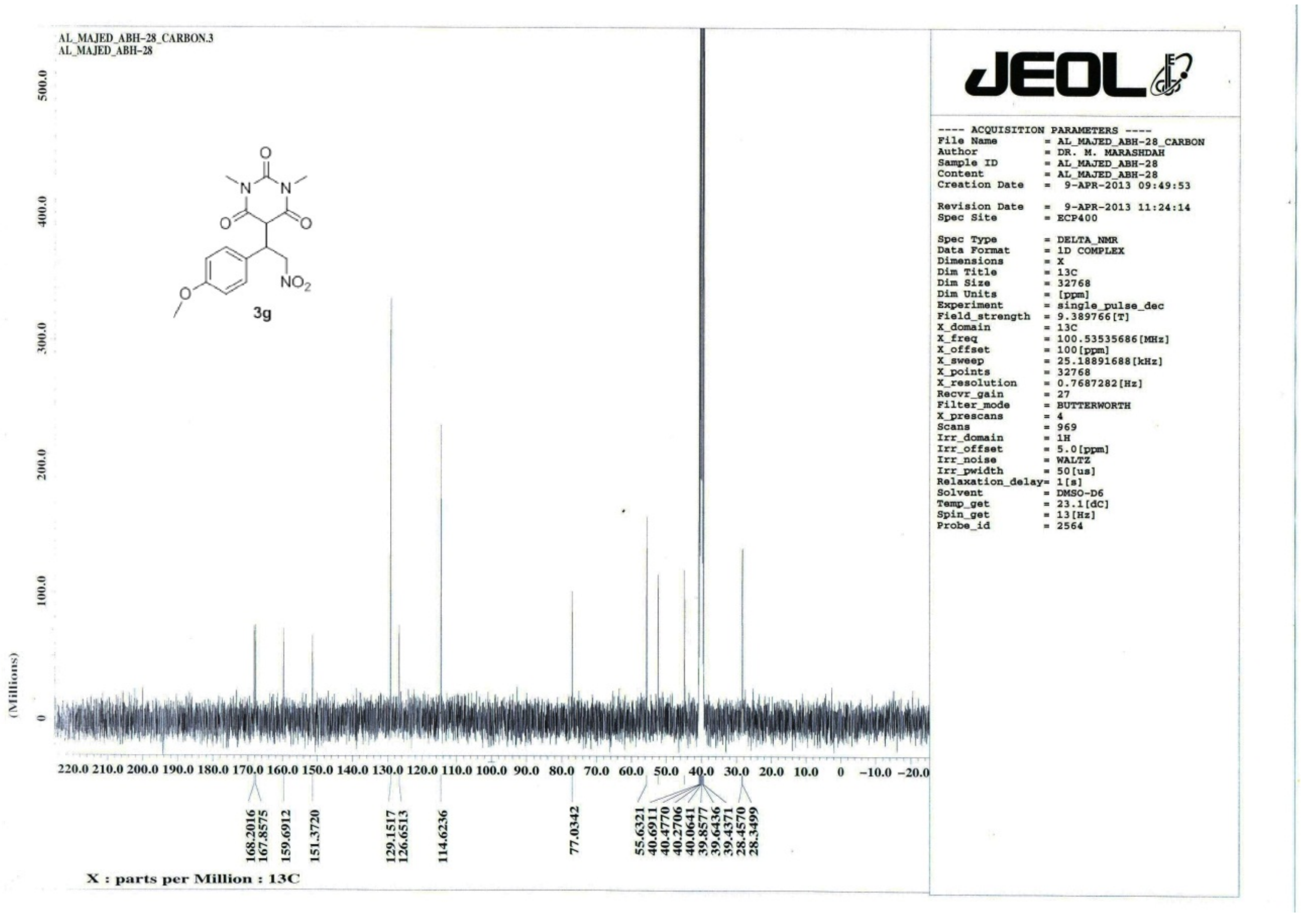

Figure S14. ¹H-NMR (DMSO-d_6) of **3h**.

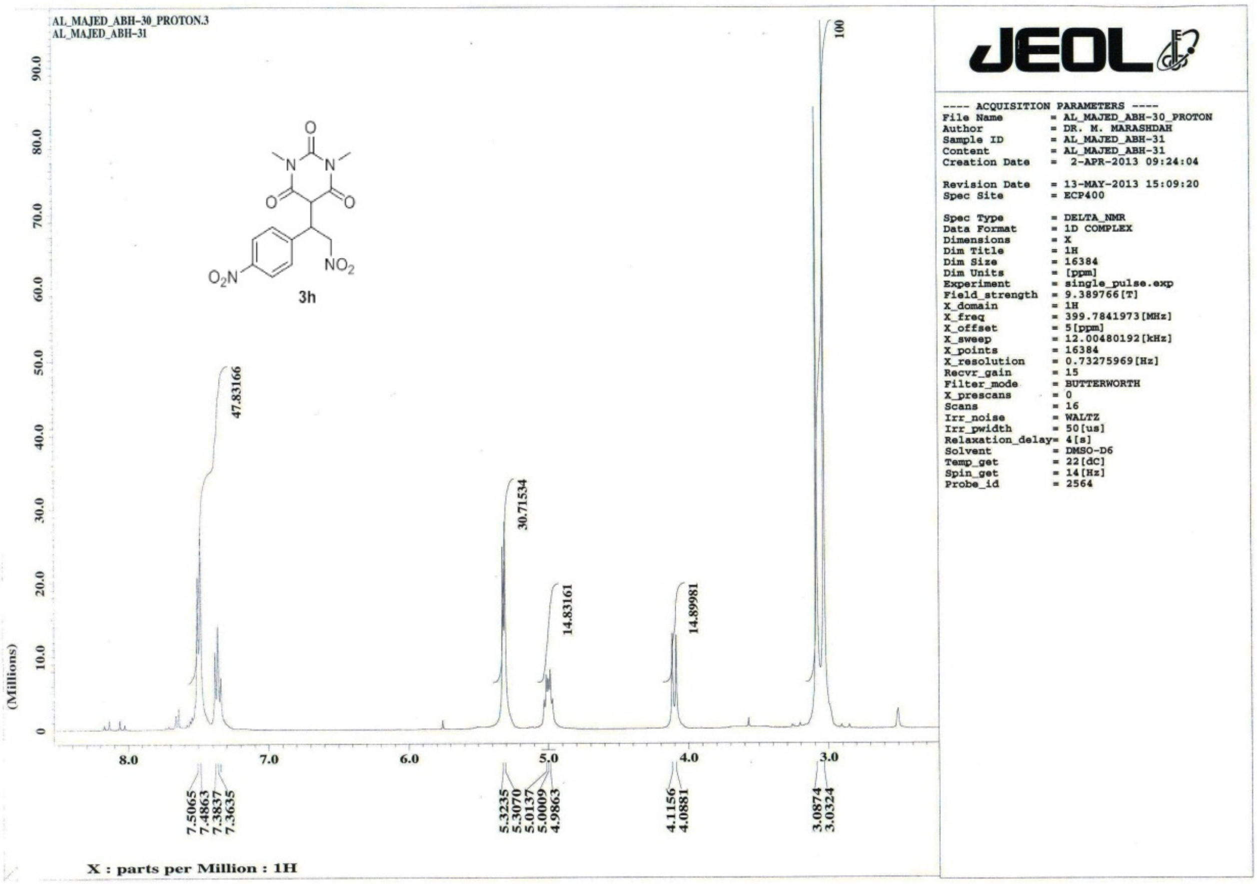

Figure S15. ^{13}C-NMR (DMSO-d_6) of **3h**.

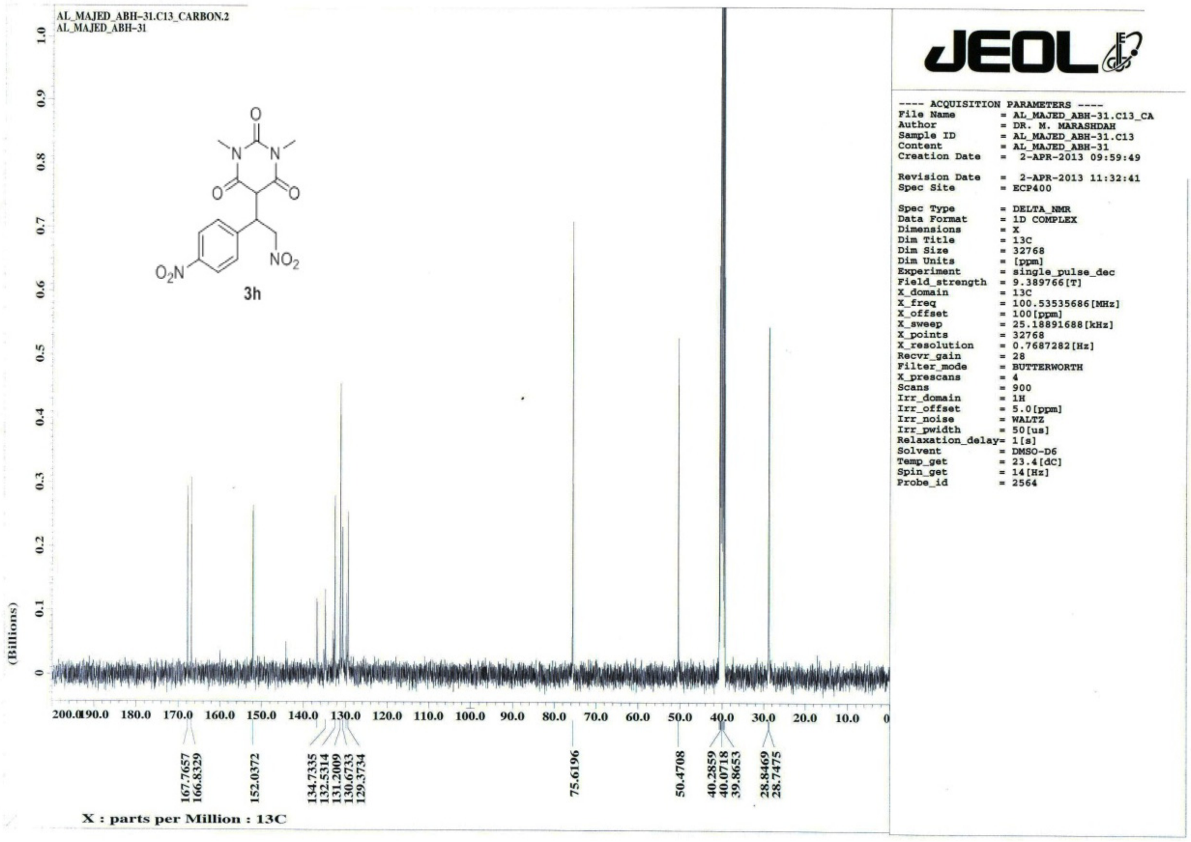

Figure S16. ^{1}H-NMR (DMSO-d_6) of **3i**.

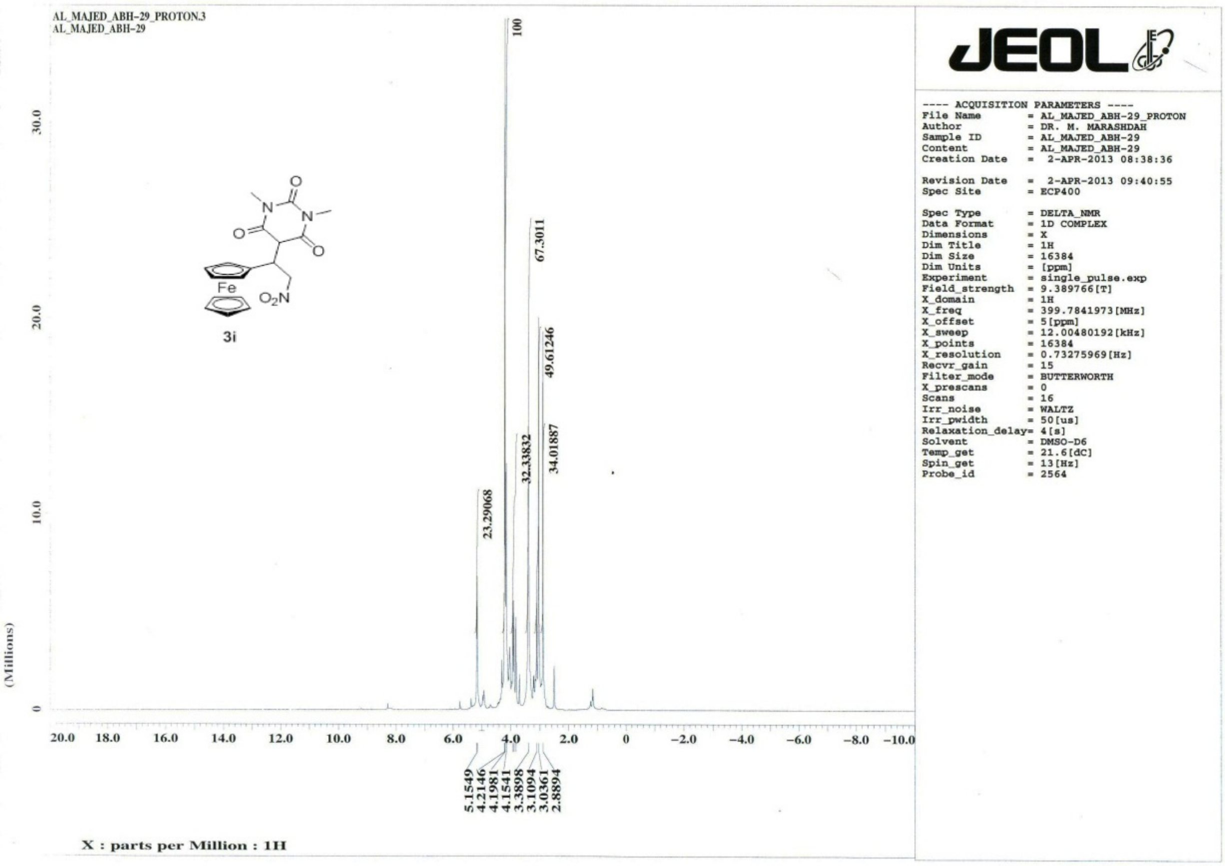

Figure S17. ^{13}C-NMR (DMSO-d_6) of **3i**.

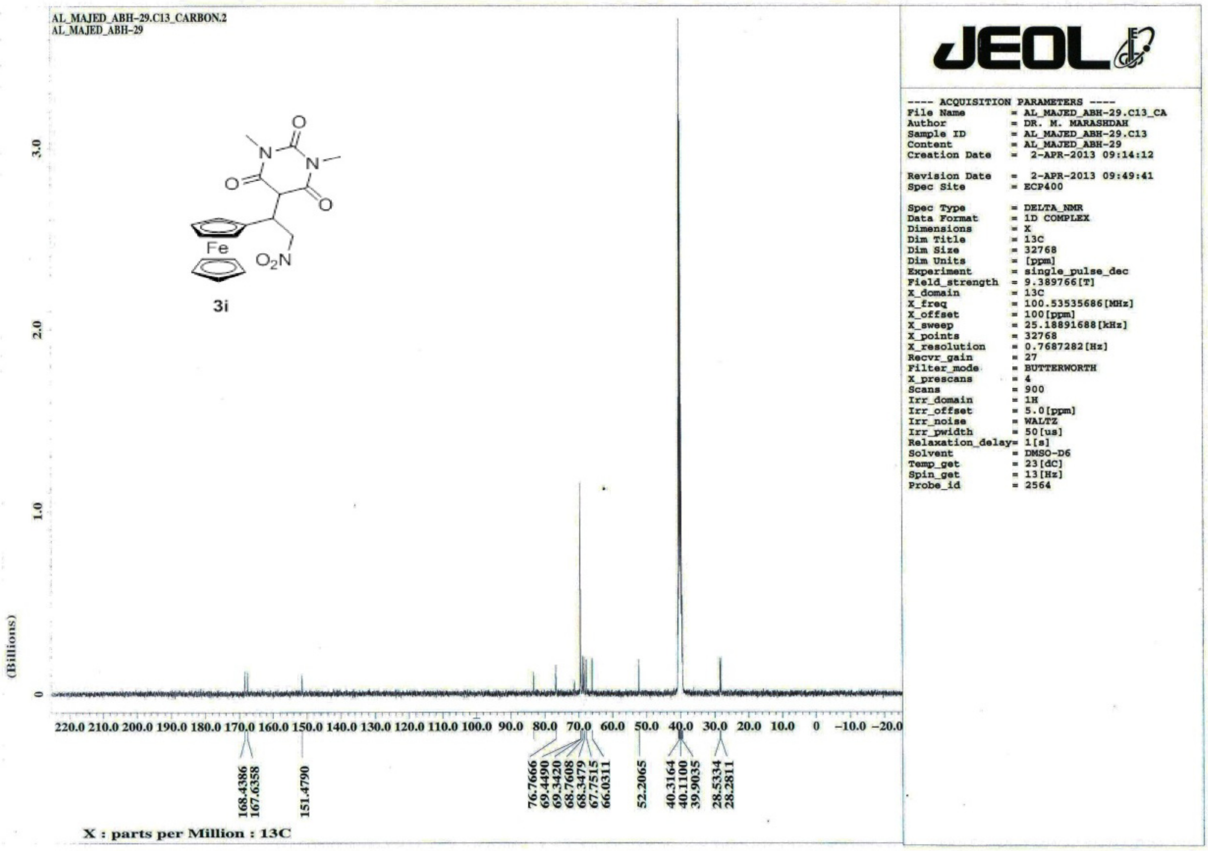

Figure S18. ^{1}H-NMR (DMSO-d_6) of **3m**.

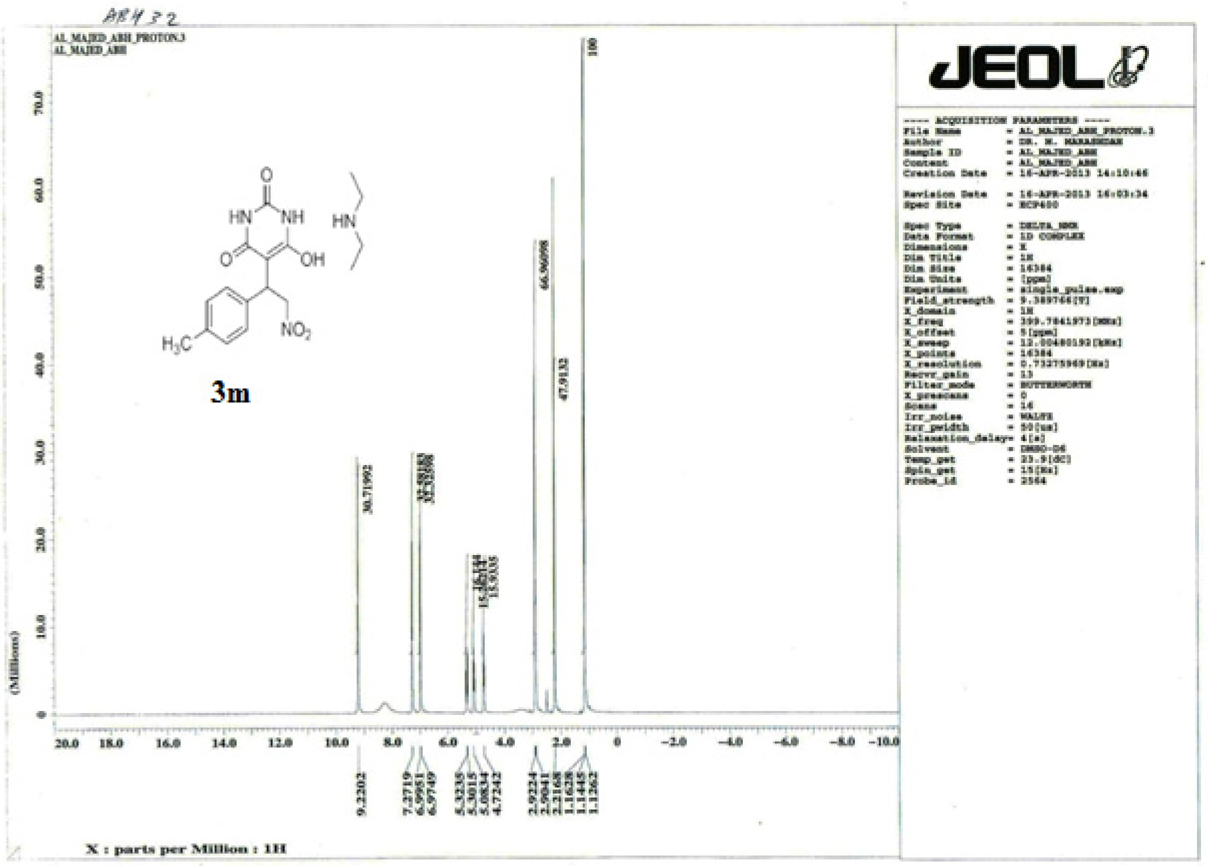

Figure S19. ^{13}C-NMR (DMSO-d_6) of **3m**.

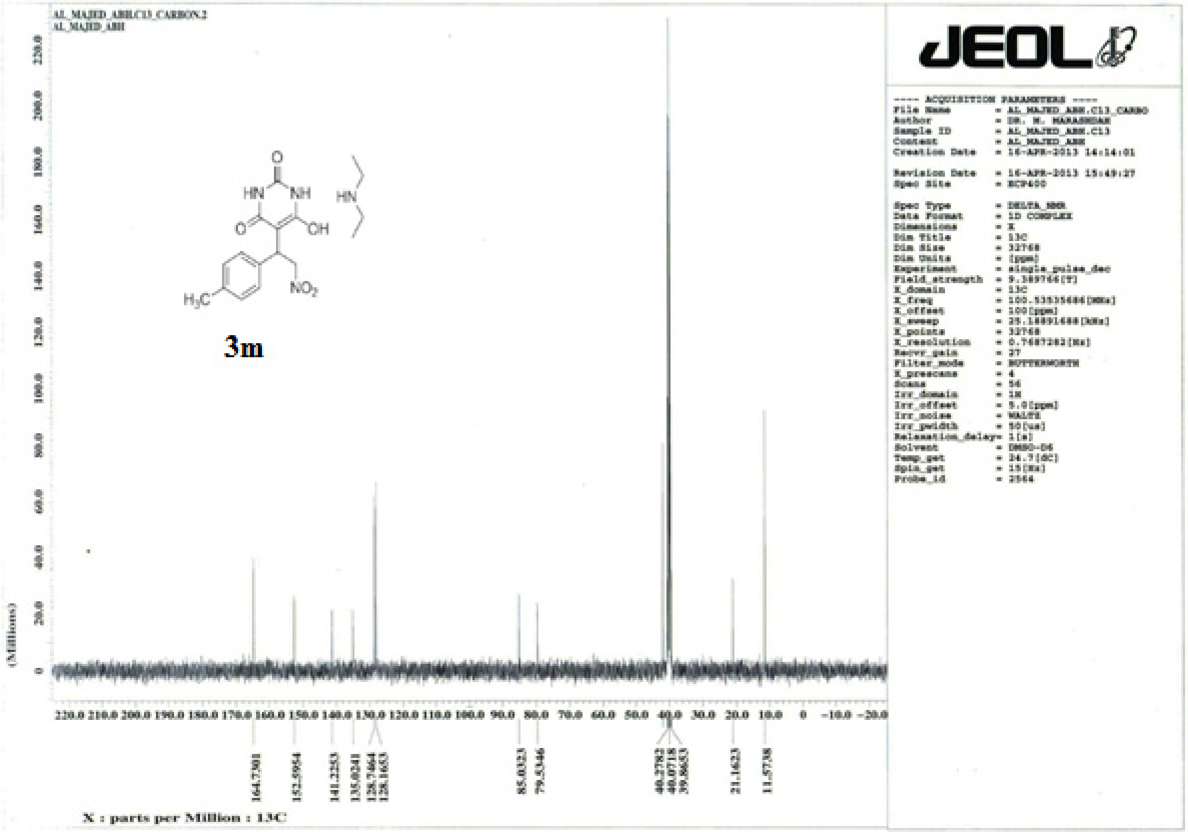

Figure S20. ^{1}H-NMR (DMSO-d_6) of **3p**.

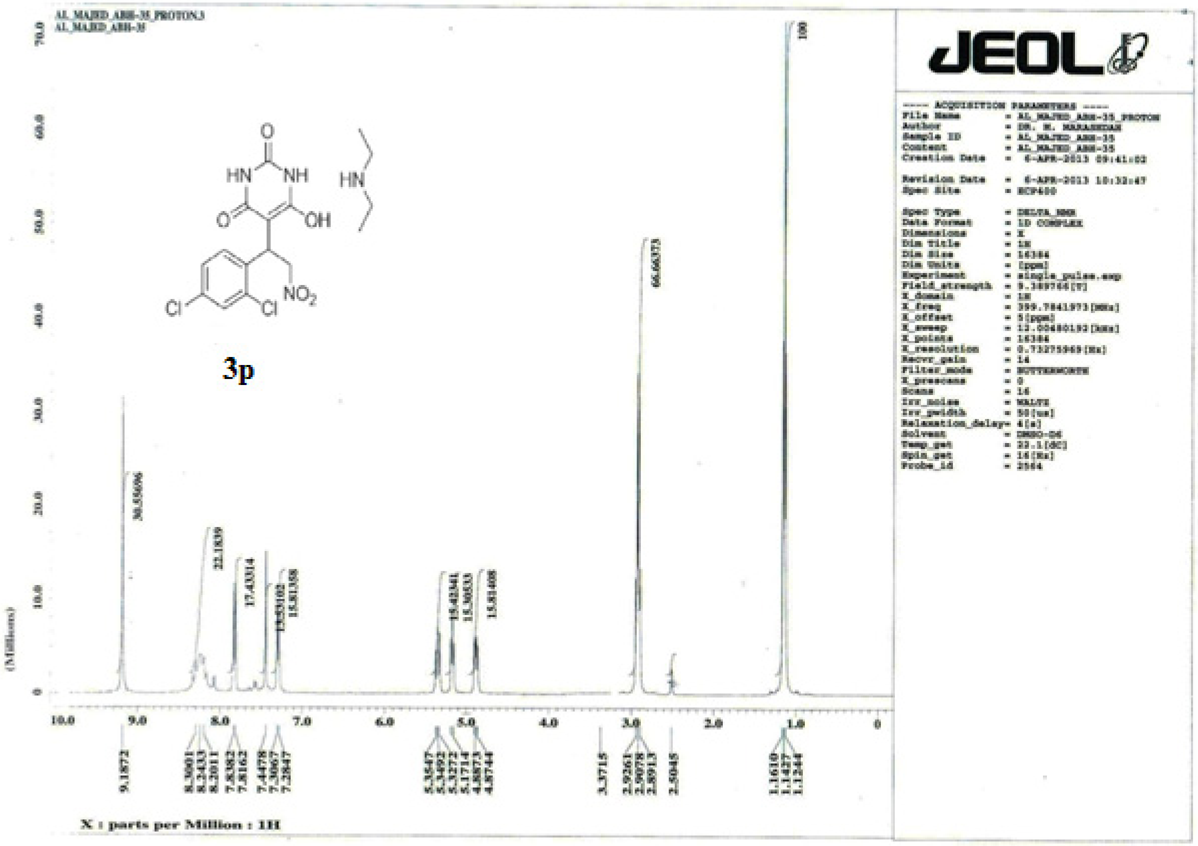

Figure S21. ^{13}C-NMR (DMSO-d_6) of **3p**.

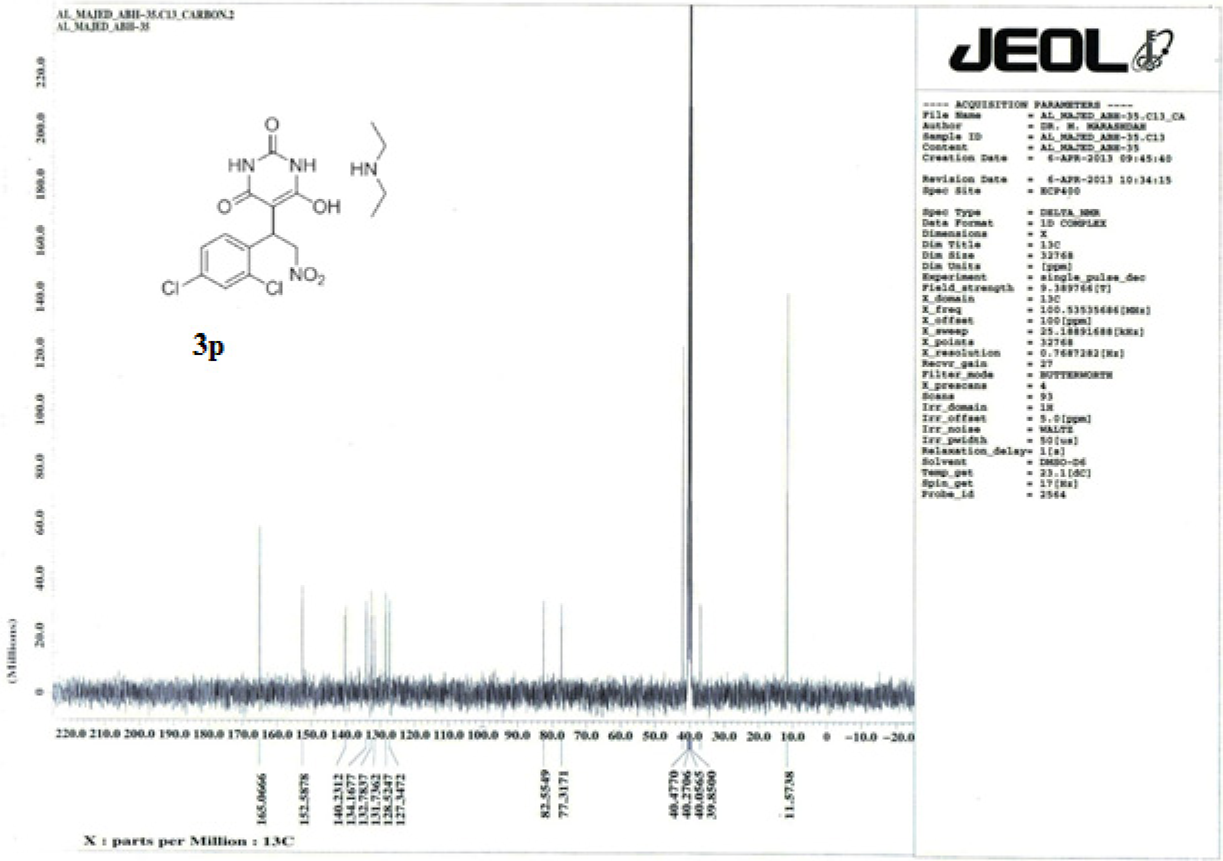

Figure S22. ^{1}H-NMR (DMSO-d_6) of **3r**.

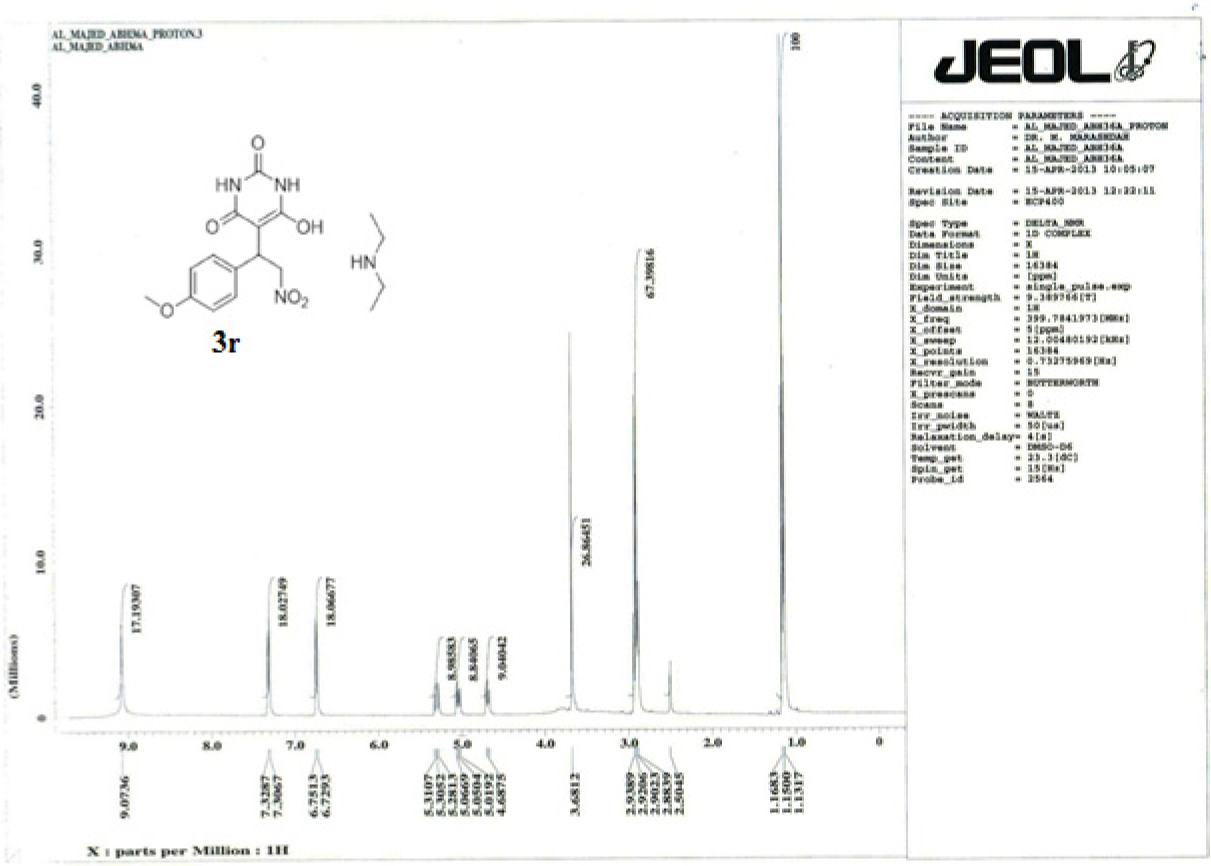

Figure S23. ^{13}C-NMR (DMSO-d_6) of **3r**.

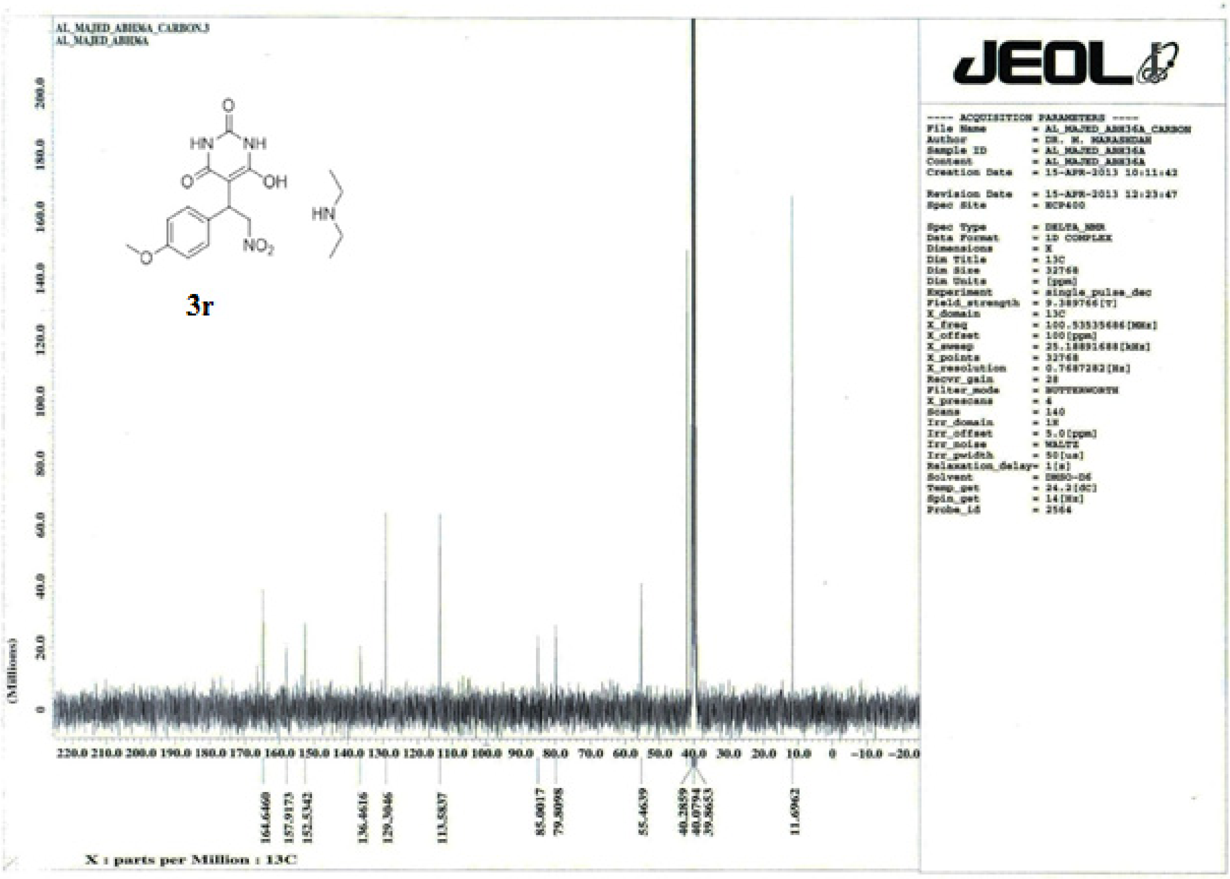

Figure S24. ^{1}H-NMR (DMSO-d_6) of **3s**.

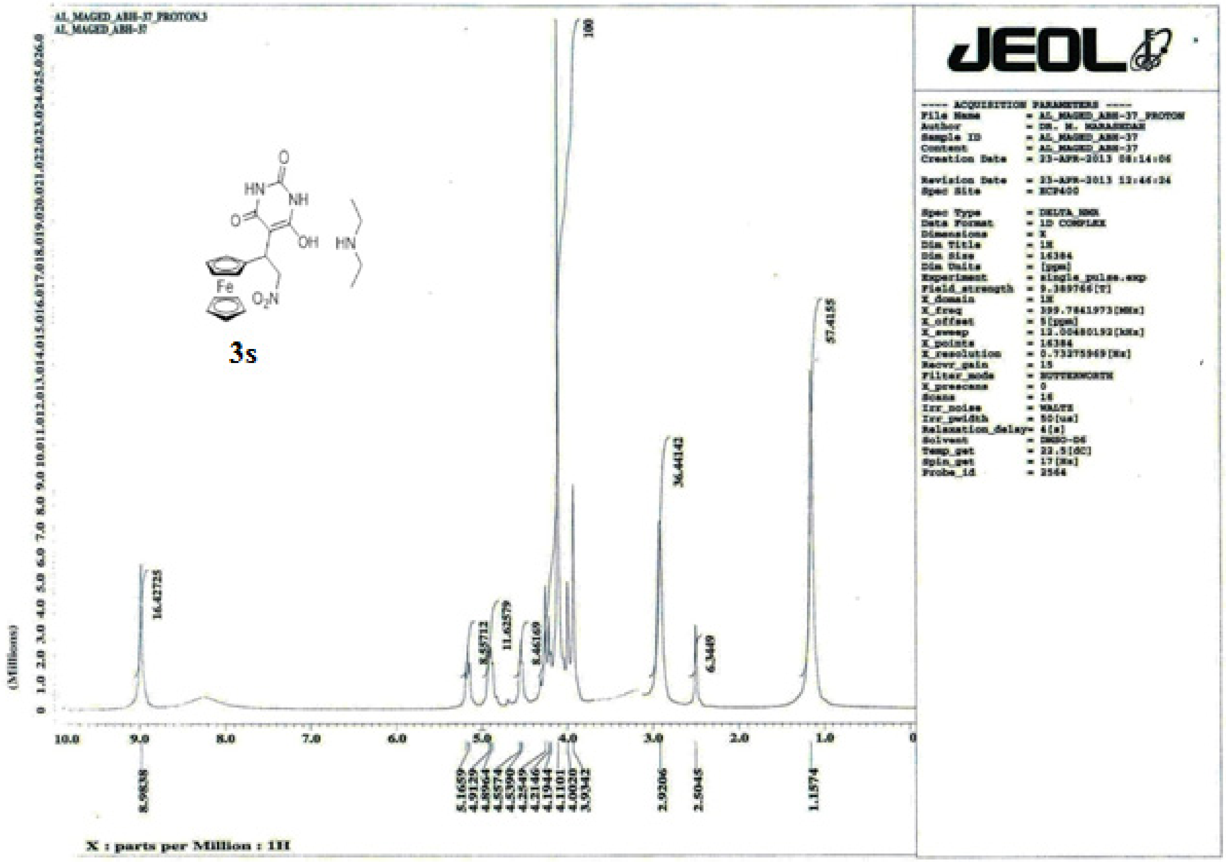

Figure S25. ^{13}C-NMR (DMSO-d_6) of **3s**.

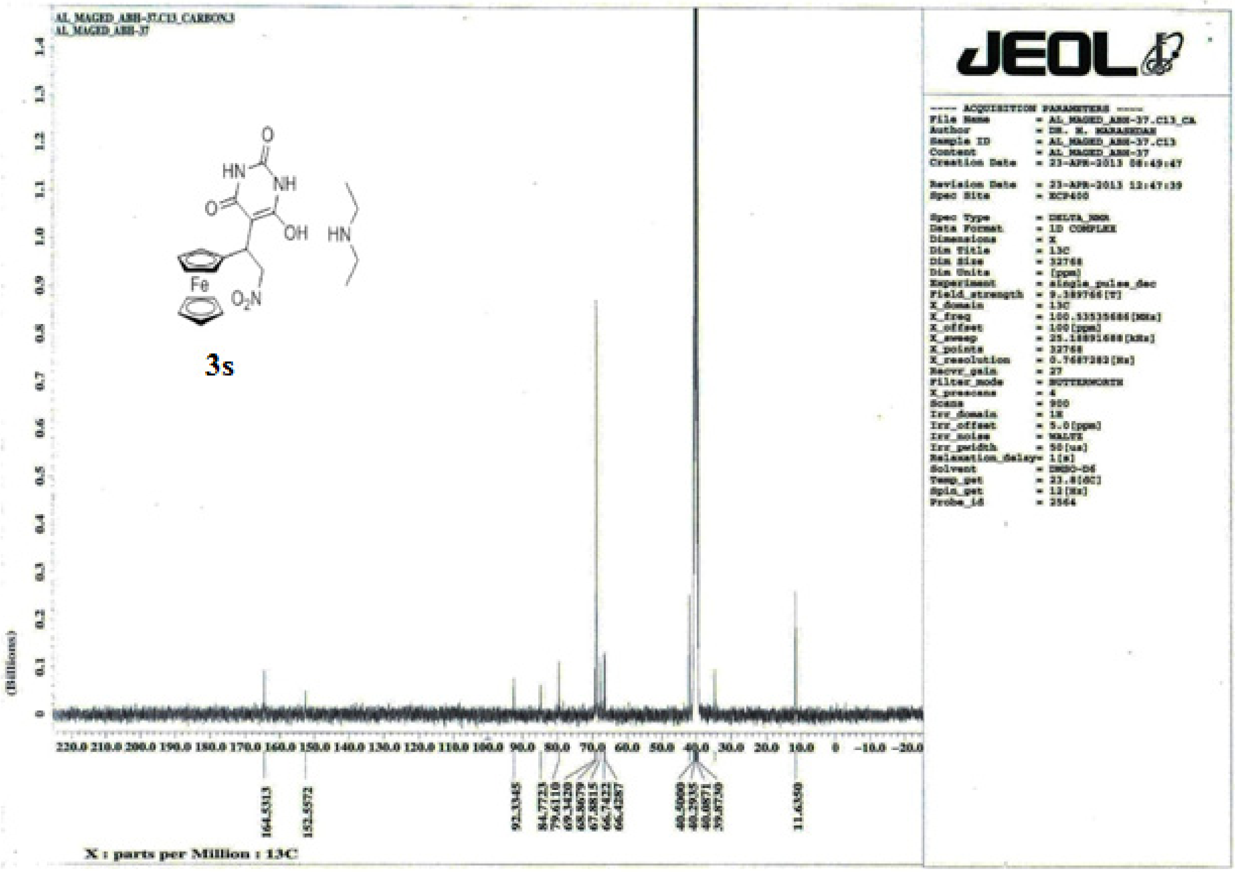

Figure S26. ^{1}H-NMR (DMSO-d_6) of **3t**.

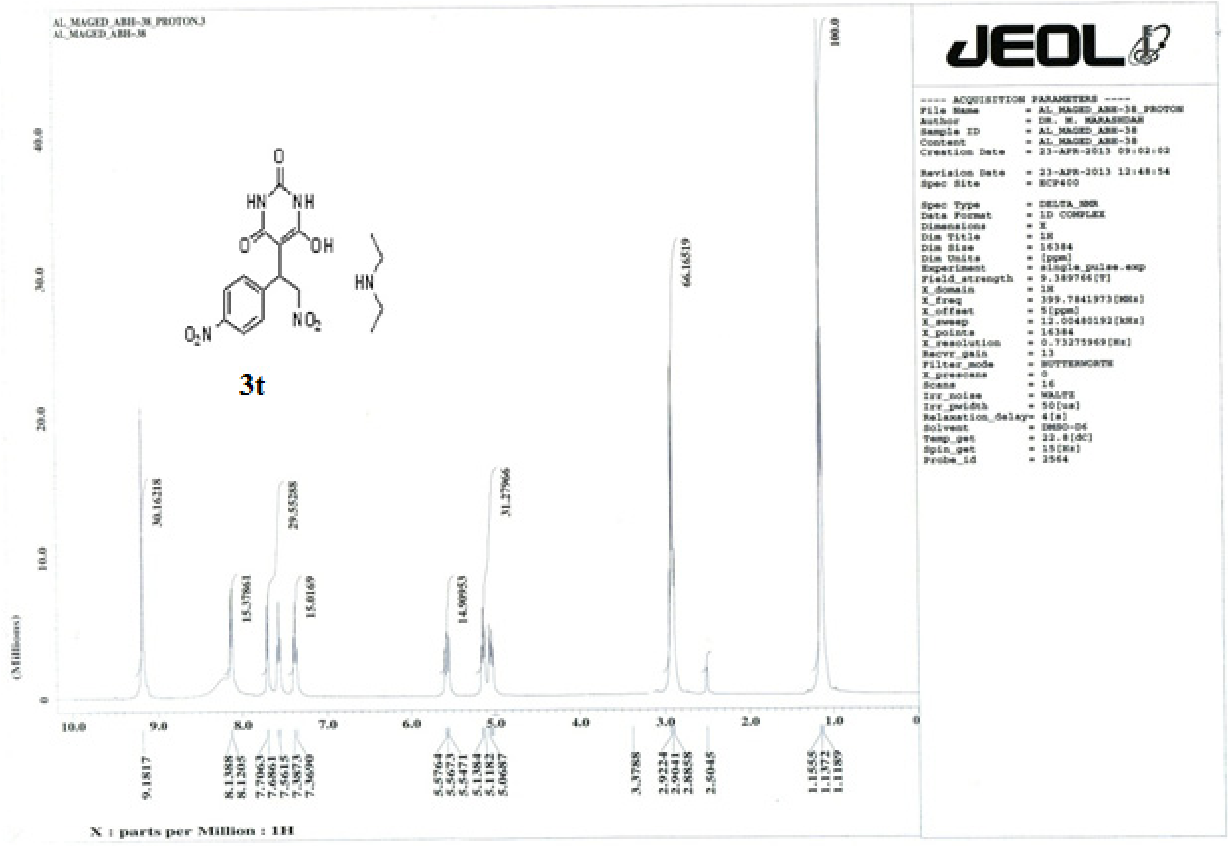

Figure S27. ^{13}C-NMR (DMSO-d_6) of **3t**.

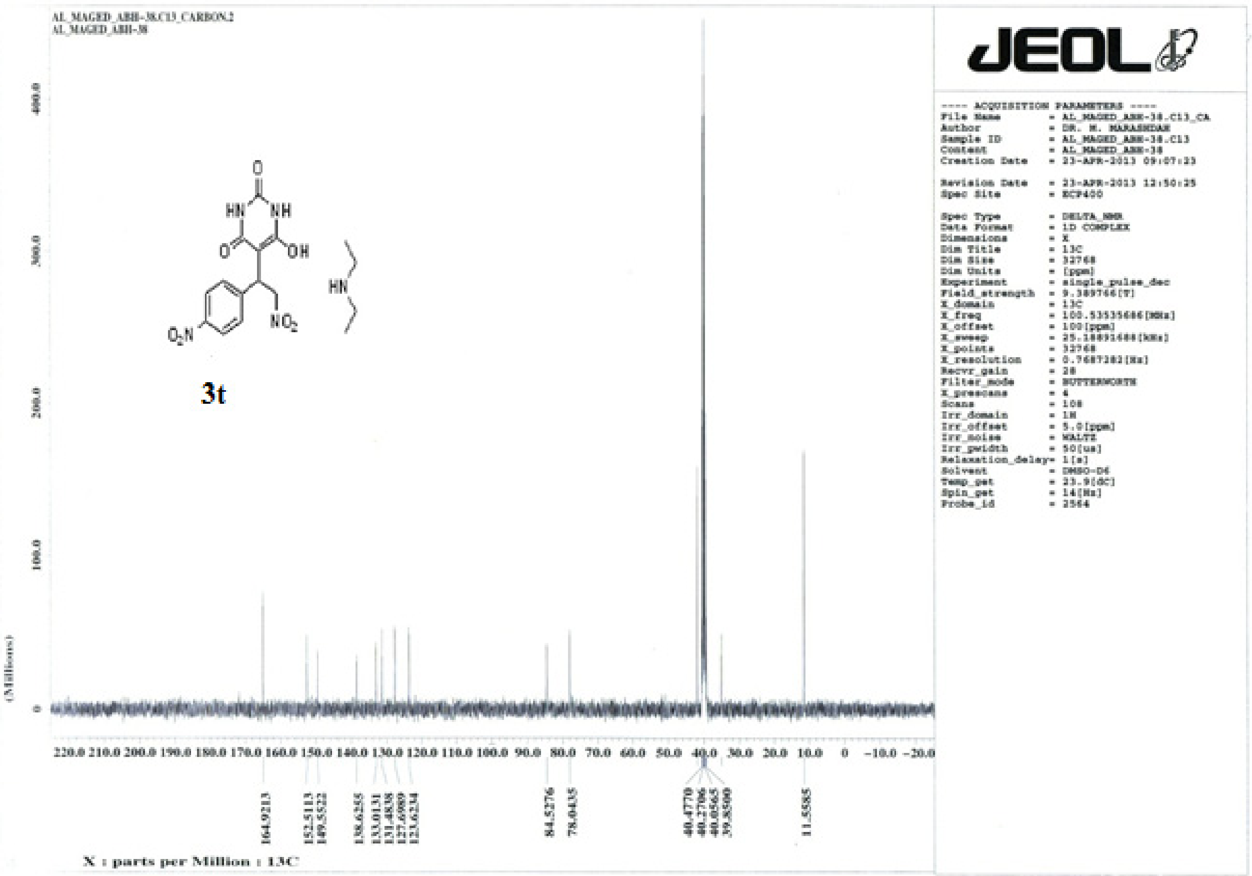

Supplement: Supplementary file 1 [file molecules-19-01150-s001.pdf]
